# CiftiStorm pipeline: facilitating reproducible EEG/MEG source connectomics

**DOI:** 10.3389/fnins.2024.1237245

**Published:** 2024-04-12

**Authors:** Ariosky Areces-Gonzalez, Deirel Paz-Linares, Usama Riaz, Ying Wang, Min Li, Fuleah A. Razzaq, Jorge F. Bosch-Bayard, Eduardo Gonzalez-Moreira, Lifespan Brain Chart Consortium (LBCC), Global Brain Consortium (GBC), Cuban Human Brain Mapping Project (CHBMP), Marlis Ontivero-Ortega, Lidice Galan-Garcia, Eduardo Martínez-Montes, Ludovico Minati, Mitchell J. Valdes-Sosa, Maria L. Bringas-Vega, Pedro A. Valdes-Sosa

**Affiliations:** ^1^The Clinical Hospital of Chengdu Brain Sciences Institute, School of Life Science and Technology, University of Electronic Science and Technology of China, Chengdu, China; ^2^School of Technical Sciences, University “Hermanos Saiz Montes de Oca” of Pinar del Río, Pinar del Rio, Cuba; ^3^Department of Neuroinformatics, Cuban Neurosciences Center, Havana, Cuba; ^4^Hangzhou Dianzi University, Hangzhou, Zhejiang, China; ^5^McGill Centre for Integrative Neurosciences MCIN, LudmerCentre for Mental Health, Montreal Neurological Institute, McGill University, Montreal, QC, Canada; ^6^Center for Biomedical Imaging and Neuromodulation, Nathan Kline Institute for Psychiatric Research, Orangeburg, NY, United States; ^7^Center for Mind/Brain Sciences (CIMeC), University of Trento, Trento, Italy

**Keywords:** human connectome project, megconnectome, Brainstorm, Ciftify, VARETA, SSSBL, HIGGS, forward model

## Abstract

We present CiftiStorm, an electrophysiological source imaging (ESI) pipeline incorporating recently developed methods to improve forward and inverse solutions. The CiftiStorm pipeline produces Human Connectome Project (HCP) and megconnectome-compliant outputs from dataset inputs with varying degrees of spatial resolution. The input data can range from low-sensor-density electroencephalogram (EEG) or magnetoencephalogram (MEG) recordings without structural magnetic resonance imaging (sMRI) to high-density EEG/MEG recordings with an HCP multimodal sMRI compliant protocol. CiftiStorm introduces a numerical quality control of the lead field and geometrical corrections to the head and source models for forward modeling. For the inverse modeling, we present a Bayesian estimation of the cross-spectrum of sources based on multiple priors. We facilitate ESI in the T1w/FSAverage32k high-resolution space obtained from individual sMRI. We validate this feature by comparing CiftiStorm outputs for EEG and MRI data from the Cuban Human Brain Mapping Project (CHBMP) acquired with technologies a decade before the HCP MEG and MRI standardized dataset.

## 1 Introduction

Over the years, electroencephalogram (EEG) and, more recently, magnetoencephalogram (MEG) have emerged as two primary techniques for non-invasively measuring brain electrical activity, offering an exceptional temporal resolution for neuroscience research and clinical applications (Da Silva, 2013). Electrophysiological source imaging (ESI) (Baillet et al., 2001; He et al., 2018, 2019, 2020), a set of techniques allowing reconstructions across the brain generators of MEG/EEG data with earliest instances in MEG (Hämäläinen and Ilmoniemi, 1994) and EEG (Pascual-Marqui et al., 1994), has found practical application in various brain imaging contexts. ESI has advanced connectomics research, offering a robust descriptive framework for all neural processes and underlying functional networks during resting state or task execution (Engel et al., 2001; Varela et al., 2001; Brookes et al., 2012, 2014, 2016; Tewarie et al., 2014, 2016, 2019a,b; Vidaurre et al., 2017; Vidaurre et al., 2018a,b).

It is widely recognized that the poor spatial resolution of inverse solutions must be, insofar as possible, overcome for multimodal neuroimaging studies, such as the study of resting-state networks employing MEG inverse solutions (Bruns et al., 2000; Tuunanen et al., 2003; Bruns, 2004; Freeman et al., 2009; Brookes et al., 2011a,b; Cabral et al., 2014; Deligianni et al., 2014; Hall et al., 2014; Maldjian et al., 2014; O’Neill et al., 2015; Tsvetanov et al., 2015; Garcés et al., 2016; Tewarie et al., 2016; Coquelet et al., 2017, 2020; Lankinen et al., 2018; Engemann et al., 2020). A successful example is finding a robust statistical relation between the power envelope of MEG inverse solutions and fMRI time series (Hipp et al., 2012).

Despite being theoretically possible, similar multimodal neuroimaging studies are more challenging to achieve when employing the EEG inverse solutions due to a more significant effect of head conductivity, which causes substantial spatial distortions (Kobayashi et al., 2003; Schoffelen and Gross, 2009; Haufe et al., 2013; Burle et al., 2015; Colclough et al., 2015, 2016; Bradley et al., 2016; Mahjoory et al., 2017; Stokes and Purdon, 2017; Palva et al., 2018; Haufe and Ewald, 2019; Marinazzo et al., 2019). This study discusses the essential factors associated with EEG ESI distortions and introduces a new pipeline facilitating EEG integration with multimodal neuroimaging and connectomics research. Although our pipeline is helpful for both EEG and MEG data, we validate the EEG ESI pipeline employing high-quality HCP MEG as a reference.

### 1.1 Challenges for reproducible ESI research within the connectomics framework

The Human Connectome Project (HCP) (Van Essen et al., 2012a,b, 2013; Glasser et al., 2013; Marcus et al., 2013) has played a pivotal role in delivering acquisition and preprocessing standards through an open-access neuroinformatic facility. This facility includes data and processing pipelines for replicable multimodal neuroimaging research comprising high-quality MEG data (Larson-Prior et al., 2013). The HCP FieldTrip megconnectome pipeline integrates MEG ESI with other HCP deliverables such as the extensively preprocessed structural MRI (sMRI) (Glasser et al., 2013), functional MRI (fMRI) (Smith et al., 2013), and diffusion MRI (dMRI) (Sotiropoulos et al., 2013).

Integrating ESI with standard neuroinformatic facilities has been central for the HCP and other global brain initiatives promoting reproducible ESI research associated with connectomics (Reid et al., 2019). This goal is affected by consistently developing and maintaining standard ESI pipelines. Noteworthy among these standardization initiatives are the Global Brain Consortium (GBC) (Valdes-Sosa et al., 2022), the United Kingdom Biobank (UKB) (Miller et al., 2016), the Healthy Brain Networks (HBN) (Alexander et al., 2017), the Helmholtz International BigBrain Analytics and Learning Laboratory (HIBALL) (Amunts et al., 2016), the Cuba Canada China Axis (CCC-AXIS) (Evans et al., 2020), and the Cuban Human Brain Mapping Project (CHBMP) (Valdes-Sosa et al., 2021). These initiatives have outlined three fundamental requirements that align with the goals of the HCP:

- Implementing ESI pipelines to achieve consistent results across diverse datasets can cope with the overwhelming number of datasets available, thus attenuating the impact of heterogeneity and incorporating automated quality control.
- Producing ESI maps in the sMRI high-resolution HCP T1w or MNINonLinear and Native and FSAverage canonical spaces. This integration must complement the data-driven or model-driven analysis of multimodal image fusion and connectomics with adequate spatial and temporal resolution.
- Producing precise cortical ESI mappings compliant with the HCP surface-based processing. This aspect crucially depends on the availability of the individual’s MRI for accurate registration and labeling, which captures the intricate neocortical structural and functional features across individuals and neuroimaging modalities.

Many existing clinical or basic neurosciences datasets, including MEG, EEG, and sMRI, arise from previously designed acquisition protocols, machines, formats, quality standards, and preprocessing. Such so-called “legacy datasets” have very diverse levels of spatial resolution. The spatial resolution can range from low-sensor-density EEG recordings without structural magnetic resonance imaging (sMRI) to high-density MEG recordings with an HCP multimodal sMRI-compliant protocol. The lack of consistent ESI pipelines that cater to such diversity is causing a significant gap in the research, hindering reproducibility. Achieving ESI consistency across diverse datasets is also essential for normative MEG/EEG procedures (Li et al., 2022). Extending this normative work on MEG/EEG sensor data to the ESI source data poses formidable difficulties with preprocessing and harmonization (Reyes et al., 2023).

In what follows, we focus on the particular ESI recommendations and consider (a) dissecting the possible quality indicators emerging from various forward and inverse modeling ingredients and (b) delivering robust forward and inverse modeling pipelines validated across sizeable databases since large sample sizes are essential to obtain high statistical power. Different ESI pipelines have been validated to consider these indicators independently, though not necessarily integrated (Bosch-Bayard et al., 2020; Bringas-Vega et al., 2022; Valdes-Sosa et al., 2022).

### 1.2 Critical forward model and inverse model ingredients for ESI

To proceed, we must conceptualize first the “forward and inverse model” terminology (applicable to both MEG/EEG ESI) and the different ingredients involved, which are the basis for obtaining inverse solutions, identifying difficulties that hinder reproducible ESI research. For an intuitive mathematical exposition, see the definitions of forward modeling (De Munck et al., 2012) and inverse modeling (Hindriks, 2020) offered elsewhere.

A *forward model*, which explains the MEG/EEG data from its brain sources, comprises three parts:

I. The source model specifies the geometry and physical nature of the MEG/EEG brain generators (Nunez and Srinivasan, 2006), e.g., a dipolar current source density model with normally oriented dipoles distributed in the vertices of the cortical mid-thickness a triangular surface mesh.
II. The head model specifies the geometry and conductivity of the different tissues across the whole volume of the head (Vorwerk et al., 2014), such as the isotropic and piecewise homogeneous volume conductor model of the head tissue, with triangular surface meshes defining the boundaries of the cerebrospinal fluid, brain, skull, and scalp tissue.
III. The lead field is the numerical approximation to an operator, also known as a Green function, which represents the integral solution to the quasistatic electric potential or magnetic field equation in the head’s medium. The lead field ranges a discrete subspace of the Green function’s domain (sources) and codomain (EEG or MEG sensors) (Hallez et al., 2007), explaining observations for the electric potential or magnetic field resulting from the current source density. This approximation, a real-valued matrix, is commonly achieved through numerical methods derived from weak integral formulations of equations, e.g., Finite Element Method (FEM) (Strang et al., 1974) or Boundary Element Method (BEM) (Cheng and Cheng, 2005).

The inverse model is designed to allow the estimation of the latent variables (sources) from the EEG/MEG data based on the given forward model. An inverse model must address the challenges associated with an ill-posed inverse problem (with no unique solution) that is further aggravated by severe ill-conditioning. Due to many more sources than MEG or EEG sensors in ESI practice, severe ill-conditioning arises from a very low-rank lead field operator. The inverse model comprises two aspects:

IV. An inverse solution method is a procedure to find an approximated solution to the Hadamard ill-posed inverse problem of electromagnetism (Hadamard, 1902). These methods are conceived within a particular mathematical framework, e.g., Tikhonov regularization (Tikhonov and Arsenin, 1977) or Bayesian statistical learning (Wipf and Nagarajan, 2009). An inverse solution in either framework is summarized by an operator (lead field pseudoinverse) minimizing a cost function for the latent current source density variables from their MEG/EEG sensor data, which also incorporates the lead field.
V. The physical and mathematical priors integrated into the possibly Bayesian cost function formalism lead to particular inverse solutions and resolve the lead field ill-condition, e.g., Tikhonov regularization function (Vega-Hernández et al., 2008) or *a priori* probabilities (Trujillo-Barreto et al., 2008). The priors may encode structural and functional information for the neural dynamics mesoscopically described by a current source density field in space, time, and frequency domains.

### 1.3 CiftiStorm: HCP FieldTrip megconnectome pipeline compliant in the Brainstorm suite

The HCP structural processing pipeline can deliver high-quality cortical segmentation and registration in the MNINonLinear/FSAverage canonical space (Glasser et al., 2013). Such high-quality segmentations may facilitate precise source model preprocessing across human individuals. The HCP structural pipeline leverages surface-based processing with computational geometry methods such as multimodal surface matching (Robinson et al., 2014). Achieving this segmentation quality requires high-contrast hybrid images from the T1 and T2 weighted (T1w/T2w) sMRIs (Van Essen et al., 2012a). Since this type of image is not always available, much effort in computational geometry has been dedicated to developing flexible structural pipelines bridging the quality gap between high-quality HCP-like segmentations (from T1/T2w sMRI datasets) and legacy segmentations from datasets containing only T1w sMRI. Essential concepts and methods on surface-based processing are included in the FSL suite (Jenkinson et al., 2012), the FreeSurfer suite (Fischl, 2012), and the Ciftify pipeline (Dickie et al., 2019), an HCP-compliant FreeSurfer bundle within the fMRIPrep pipeline (Esteban et al., 2019).

Achieving precision in the ESI analyses is predicated on having realistic lead fields (Piastra et al., 2020). However, lead field calculations, rigorously thought, involve tackling the solution for the electric potential or magnetic field equation for a highly detailed volume conductor model of the head’s medium. This model, which must be specified within numerical methods as a conductivity tensor field in a very high-resolution three-dimensional (3D) tetrahedral mesh, represents the anisotropic and heterogeneous properties of the head tissue (Nolte and Dassios, 2005; Dannhauer et al., 2012). The first problem is uncertainty, which specifies conductivities at every point in this mesh. The second is computational cost and numerical instability of the lead field methods in the complex individual geometry (Windhoff et al., 2013; Vorwerk et al., 2018; Piastra et al., 2020).

Henceforth, numerical instability refers to issues encountered in the calculation of the formulation matrix for solving partial differential equations in weak integral form, a method commonly associated with the FEM. The “formulation matrix,” or head model matrix in the context of EEG/MEG lead field computations, is inversely proportional to the squared distances and directly proportional to the conductivity differences at each point within the high-resolution 3D tetrahedral mesh. This mesh, derived from discretizing the space, aims to accurately represent the physical properties, such as conductivity, within a complex geometry (individual head). Unfortunately, several numerical challenges arise when calculating the adjoint of a formulation matrix, including issues such as low precision, scaling discrepancies, and ill-conditioning. Addressing these problems may require exhaustive procedures, particularly in cases of intricate geometries.

Therefore, a moderately detailed volume conductor model of the head tissue may be assumed to alleviate the computational cost, numerical instability, and uncertainty associated with lead field calculations. These models consider isotropic and piecewise homogeneous conductivities of the significant head compartments, with enclosed boundaries defined by high-resolution 3D triangular meshes. However, numerical instability might persist during the calculations of the adjoint matrix associated with the BEM (Hamalainen and Sarvas, 1989; Hämäläinen et al., 1993; Riera and Fuentes, 1998).

This situation poses a significant challenge, especially when dealing with large EEG datasets, as these issues strain the computational capacity and currently available quality control strategies. Consequently, many opt for alternative approaches, such as average forward models (Litvak et al., 2011; Ashburner et al., 2014), spherical forward models (Rush and Driscoll, 1969; Schneider, 1974; Roth et al., 1993), or homogeneous forward models (Nunez, 1974; Jirsa and Haken, 1997; Nunez and Srinivasan, 2006), to mitigate these computational and numerical challenges.

The megconnectome pipeline is a successful effort for ESI integration within the HCP research framework (Larson-Prior et al., 2013) that employs the FieldTrip suite (Oostenveld et al., 2011). However, megconnectome is tightly framed within HCP MEG standard format, FSAverage source space modeling, magnetic head and lead field modeling, and inverse modeling approaches. Unfortunately, several restrictions prevent the direct extension of the megconnectome pipeline for forward or inverse modeling with diverse MEG or EEG datasets.

The Brainstorm suite (Tadel et al., 2011) was produced through a broader collaborative effort to set up unrestrictive and easy-to-use interfaces to design neuroinformatic tools and pipelines for ESI from scratch. Brainstorm has readily incorporated an inclusive neuroinformatic ecosystem that includes structural pipelines and forward and inverse modeling pipelines for diverse acquisitions, including not only for MEG/EEG but also electrocorticogram (ECoG), intracranial EEG (iEEG), and functional near-infrared spectroscopy (fNIRS). This ecosystem includes and integrates some of the most noteworthy neuroinformatic software, such as SPM12, FieldTrip, OpenMEEG, and DUNEuro.

We aim to expand efforts within the HCP ESI research framework, developing CiftiStorm, a megconnectome-compliant pipeline based on the Brainstorm suite. The only available Brainstorm pipeline sharing similar functionality to the megconnectome pipeline is the reproducible analysis pipeline applied to resting-state MEG data from the Open MEG Archive (OMEGA) (Niso et al., 2019). No other pipeline with the same purpose as CiftiStorm fulfills the ESI requirements (Section 1.1). FieldTrip might be an alternative to Brainstorm in devising our pipeline, an aspect we are currently evaluating.

We also provide novel tools for analyzing functional connectivity data for sensors and sources time series in the frequency domain. Specifically, we implement inverse methods to estimate the source cross-spectral tensor. This tensor is a 3D array (sources × sources × frequencies) in which each frontal slice, a 2D array (sources × sources), is the complex-valued Hermitian covariance matrix of the Fourier coefficient. This matrix, an estimator for the second-order statistical moment of the multivariate probability distribution of the Fourier coefficients, summarizes all the statistical properties for oscillatory brain networks under stationarity and mixing conditions that apply to brain activity during resting state or task execution in a current source density. Under these conditions, the probability distribution of the Fourier transform converges asymptotically to the multivariate complex-valued Gaussian distribution (Brillinger, 1983). This property is akin to principles found in classical statistics, particularly those related to the central limit theorems of real-valued random variables. In a previous study (Paz-Linares et al., 2023a), a statistical test for the complex-valued Gaussian distribution produced positive results across the entire frequency spectrum of MEG and ECoG example data.

When developing CiftiStorm, we considered all the critical aspects of forward and inverse modeling described in Section 1.2. As a consequence, and as we demonstrate below, CiftiStorm produces compatible ESI results for Human Connectome Project (HCP) MEG and Cuban Human Brain Mapping Project (CHBMP) EEG across the entire spectrum in the resting state condition.

## 2 Materials and methods

**Figure 1:**
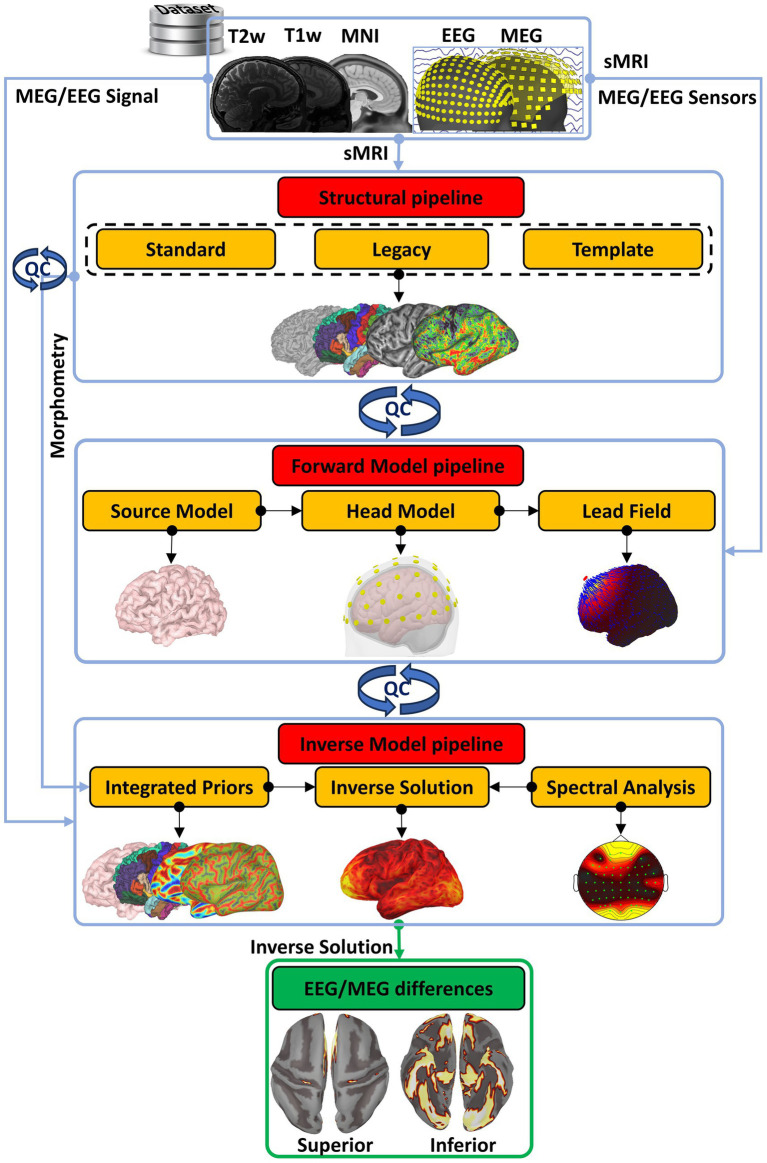
The conceptual organization of CiftiStorm. Red boxes highlight the conceptually different pipelines, and yellow boxes highlight the pipeline modules within CiftiStorm. In the top row is the definition for Brainstorm inputs within a dataset, which may include individual T1w, T2w, or none of these sMRI acquisitions; the MNI-registered sMRI template that better describes the dataset; and the EEG or MEG acquisitions. The modules include the standard and legacy modules of an HCP-compatible structural processing pipeline on the second row, the source model, head model, and lead field modules of a forward model processing pipeline on the third row, and the prior’s integration and inverse solution modules of an inverse model processing pipeline at the fourth row. CiftiStorm incorporates quality control and corrections (blue looped arrows) driven by stringent factors derived from processing by the different pipeline modules. Within the scope of our study is the production of highly similar EEG/MEG inverse solutions, illustrated in the green box, ensuring broader EEG ESI integration.

We now describe the CiftiStorm pipeline, which comprises three pipeline modules (Figure 1): structural processing (Section 2.1) based on the HCP T1/T2w sMRI or the Ciftify T1w sMRI pipeline; forward model processing (Section 2.2) includes geometrical and numerical refinements based on our previous work on lead field quality control (Riaz et al., 2023); and inverse model processing (Section 2.3) includes our Bayesian statistical learning ESI (Paz-Linares et al., 2023a,b), incorporating geometrical and dynamical priori information in the frequency domain. The inverse model/solution further develops, in terms of Bayesian algorithms and priors, the methodology of variable resolution electromagnetic tomographic analysis (VARETA) (Valdes-Sosa et al., 2000; Bosch-Bayard et al., 2001).

To ensure seamless compatibility with the Human Connectome Project (HCP) pipeline environment, we propose establishing a well-configured neuroinformatic environment for CiftiStorm. The recommended system is a 64-bit Linux operating system capable of building all necessary software, with a preference for CentOS/RHEL 7. Alternatively, CentOS/RHEL 8/9 or Fedora 38/39 can be considered. Environment specifications entail Python 2.7 and 3.7, including the essential neuroimaging-in-python libraries. The required software comprises FreeSurfer 6/7, FSL 6/7, HCP pipelines, Ciftify, Connectome Workbench, MATLAB, and Brainstorm toolbox. Additionally, ensure the necessary plugins for the SPM, FieldTrip, and EEGLAB toolboxes. Minimal hardware specifications encompass 16GB RAM, a quad-core CPU, and an 8GB GPU, which expedites the eigendecomposition loop within the inverse model pipeline. Under these conditions, the processing of a case—encompassing sMRI T1w/T2w structural processing, EEG 10–20 system forward model processing, and 8 k sources inverse model processing–can be completed within 8 h.

### 2.1 CiftiStorm structural pipeline

#### 2.1.1 Standard

The HCP structural processing pipeline (Glasser et al., 2013) and the FreeSurfer suite (Fischl, 2012) implement accurate brain segmentations, morphometrics, and labeling across individuals. HCP structural processing consists of three main bash modules (“.sh” scripts) that leverage critical FreeSurfer and FSL utilities:

- The PreFreeSurferPiepeline.sh module generates an undistorted “native” structural volume space for each subject, the T1w/T2w sMRI high-contrast hybrid image in MNI space (Evans et al., 1993), which is critical for high-quality segmentation in the subsequent modules. This module involves preprocessing, bias field correction, linear transformation (alignment), and registration (voxel-wise) of the T1w and T2w sMRI.
- The FreeSurferPipeline.sh module, a recon-all pipeline-compliant, performs segmentation of the brain structures from the high-contrast hybrid image. Subcortical and cortical structures, such as the white, mid-thickness, pial surface (triangular meshes), and thickness volume (tetrahedral mesh), are extracted during the segmentation. This stage, which involves non-linear transformation and registration to the FSAverage canonical space, produces the essential surface-based and volume-based outputs (structures, morphometrics, and labeling) mapped into the T1w/FSAverage (registered) and MNINonLinear/FSAverage (non-linearly transformed and registered) spaces.
- The PostFreeSurferPipeline.sh module produces similar surface-based and volume-based outputs mapped into the T1w/Native and MNINonLinear/Native. These outputs are produced by reversing the previous non-linear transformation and registration to the T1w/Native and MNINonLinear/Native spaces. This module also generates the Conte69 registered mesh (Van Essen et al., 2012a,b), downsampled meshes for connectivity analyses, creates the final brain mask, and produces myelin maps. The surface spaces are delivered in three different resolutions (Glasser et al., 2013): the native mesh for each individual (~136 k vertices), the high-resolution Conte-69 registered standard mesh (~164 k vertices), and the low-resolution Conte69 registered standard mesh (~32 k vertices).

The new certified data format CIFTI, designed to accommodate the geometric processing and outputs of the HCP pipelines, is a considerable upgrade from its predecessors in NIFTI (Jenkinson, 2005) and GIFTI (Harwell et al., 2008). This format harmonizes volume-based (subcortical) and surface-based (cortical) structural or functional data, unifying their coordinate systems for the volume-voxel and surface-node locations across all spaces, called grayordinates. Moreover, CIFTI is optimized to facilitate seamless cross-platform compatibility for the HCP pipelines, particularly for matrix and tensor operations, as well as read and write disk access.

#### 2.1.2 Legacy

We also include, as an alternative, the HCP-compliant structural workflow of the Ciftify pipeline (Dickie et al., 2019). CiftiStorm uses this workflow to produce HCP-compliant outputs in two situations: when the T2w sMRI is not available and when the T1w sMRI is not available or usable. In the last situation, CiftiStorm leverages the MNI-ICBM152 (Fonov et al., 2011) or MNI-AutoReg (Collins et al., 1994) T1w sMRI template to produce HCP-compliant structural outputs. CiftiStorm includes two bash modules (“.sh” scripts), freesurfer_recon_all.sh and ciftify_recon_all.sh, that can be applied to a complete dataset and obtain the structural outputs of each participant:

- freesurfer_recon_all.sh: Wrapper script that invokes the “recon-all” command line functionality. This functionality is the standard FreeSurfer pipeline, a minimal sequence of steps in the structural processing of T1w sMRI. In this stage, cortical and subcortical structures and morphometrics are produced in the T1w/FSAverage and MNINonLinear/FSAverage spaces.
- ciftify_recon_all.sh: Wrapper script that invokes the “ciftify_recon_all” command line functionality. In this stage, HCP-compatible outputs are produced in all the spaces: T1w/Native, T1w/FSAverage, MNINonLinear/Native, and MNINonLinear/FSAverage.

**Figure 2:**
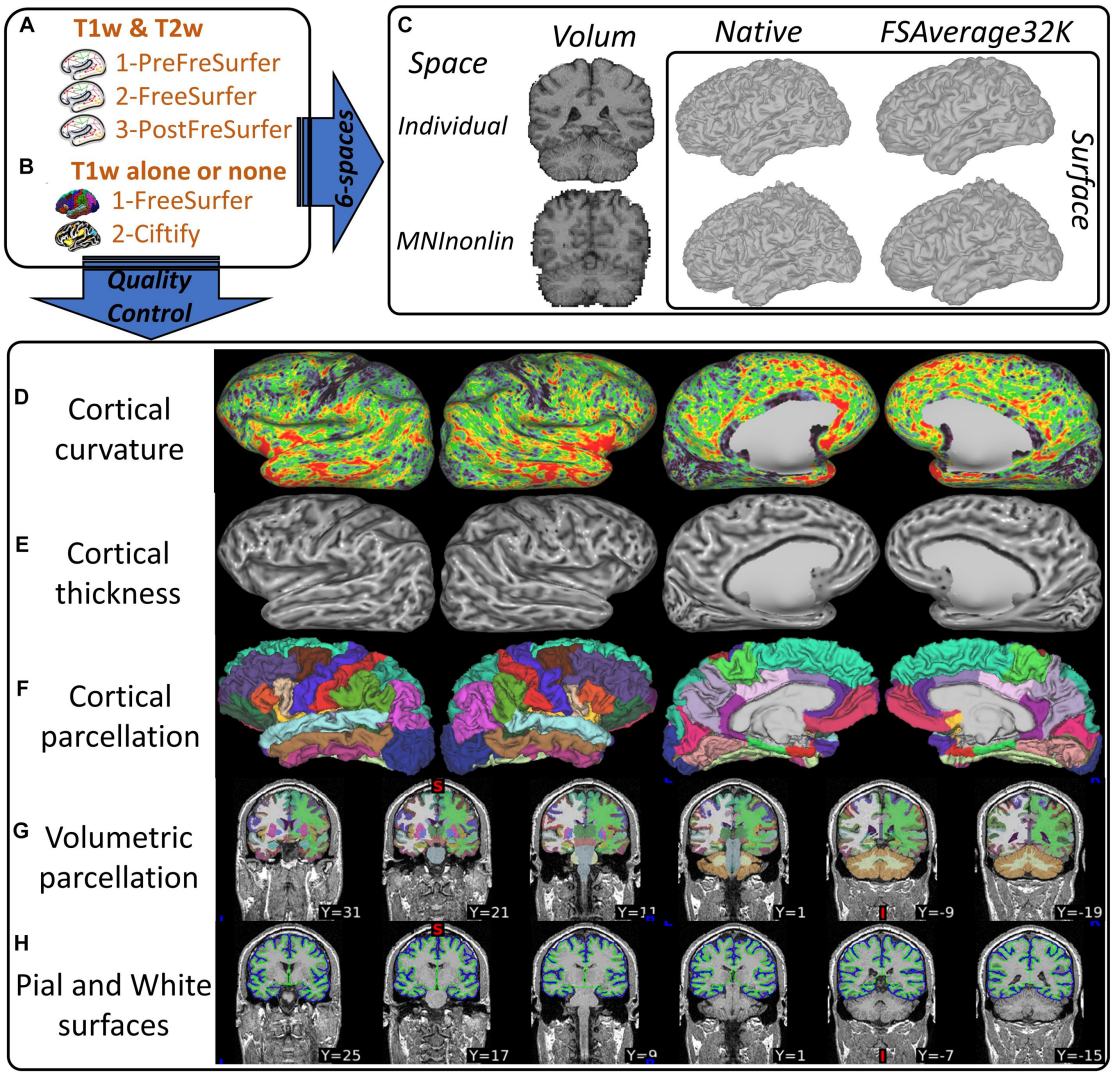
Illustration of the CiftiStorm structural processing pipeline. Top row: **(A)** the HCP standard PreFreeSurfer, FreeSurfer, and PostFreeSurfer pipeline modules that are applied to T1/T2w sMRI, **(B)** the HCP legacy FreeSurfer and Ciftify pipeline modules that are applied to T1w or sMRI (legacy databases), and **(C)** HCP spaces (volumetric and superficial) in the T1w (MNILinear) or MNINonlinear, and Native or FSAverage32k. Bottom row: **(D)** cortical curvature, **(E)** thickness, **(F)** parcellation, **(G)** volumetric subcortical segmentation, and **(H)** cortical layers mid-thickness and pial.

Figure 2 illustrates the essential HCP-compatible structural deliverables from processing T1/T2w standard data (a) and T1w or T1w-template legacy data (b). The deliverables include two volumetric outputs and four surface outputs (Figure 2C) leveraged for volume-based and surface-based processing of functional images (MEG/EEG, fMRI), morphometrics (Figures 2D,E), parcellations (Figures 2F,G) used to implement priors modeling, and cortical layers (Figure 2H).

The HCP surface-based processing and registration pipeline ensures high-quality cortical feature extraction, denoising, and comparable interindividual mapping to the MNINonLinear and FSAverage spaces. Due to the layered cortical organization (Dale et al., 1999), this type of processing exhibits greater accuracy than the volume-based approach in extracting structural features (Coalson et al., 2018) or functional features (Anticevic et al., 2008). In addition, the surface-based registration has improved cortical alignment, across all HCP spaces, individuals, and datasets. This improved alignment has increased the statistical power of tests on cortical morphometrics, from sMRI, dMRI, fMRI (Brodoehl et al., 2020), and MEG/EEG inverse solutions.

### 2.2 CiftiStorm forward model pipeline

#### 2.2.1 Source model

**Figure 3:**
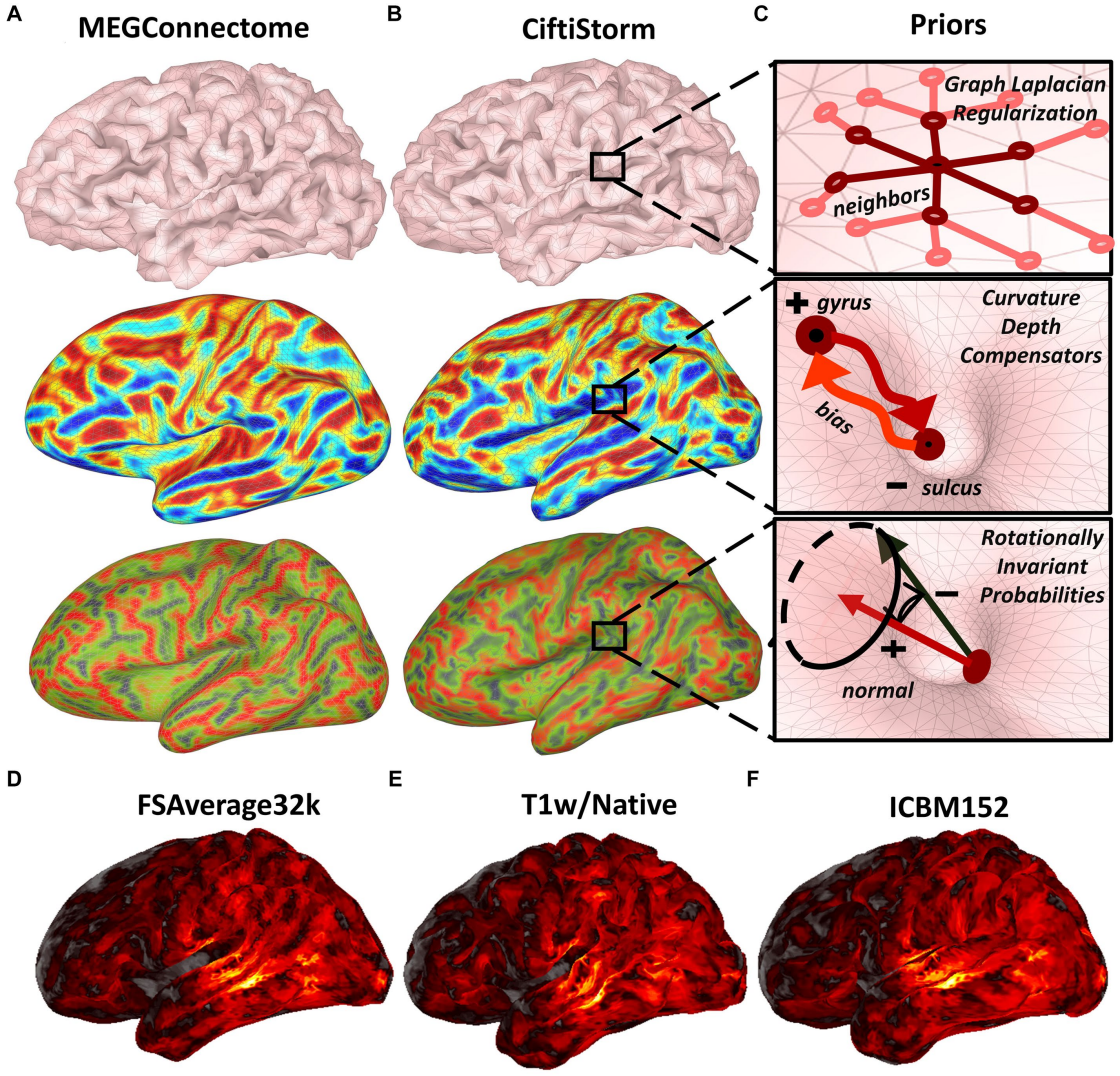
Megconnectome or CiftiStorm forward and inverse model processing leverage the surface-based source model and metrics. **(A)** The geometry includes triangular meshes (top), curvature or second-order surface metric (middle), and normal or first-order surface metric projected in the Z-axis (bottom). **(B)** Surface-based geometrical information, including the discrete Laplacian prior (top), curvature depth compensation prior (middle), and rotational invariance prior (bottom), are to be considered by inverse modeling methods. **(C)** Shows the prior representation included in our strategy for the source processing module, Graph Laplacian (top), curvature depth compensation prior (middle), and rotational invariance (bottom). In addition, a typical inverse solution was registered across all HCP spaces and interpolated to high resolution by the CiftiStorm pipeline. The inverse solution is shown in **(D)** the FSAverage32k canonical space, **(E)** the T1w/Native space, and **(F)** the MNI-ICBM152 space.

CiftiStorm ESI follows the surface-based source model processing approach in compliance with the HCP megconnectome pipeline (Larson-Prior et al., 2013) and fMRI pipeline (Smith et al., 2013). A benefit of the surface-based approach is the precise registration across individuals and modalities of the intricate cortical structural and functional features (Glasser et al., 2016). A surface-based source model assumes that cortical MEG or EEG generators may exist on the vertices of a triangular surface mesh. This model is more specific than volume-based models when describing the MEG and EEG neurophysiological origin (Nunez, 1974; Freeman, 1975; Nunez and Srinivasan, 2006), thus increasing ESI precision. Note that the user may specify in this model whether the orientation of the sources is free or constrained (see Section 2.3.2). Figure 3 illustrates the CiftiStorm and megconnectome surface-based source model and important morphometrics.

Indeed, the megconnectome individual source model outputs in the T1w FSAverage32k space are defined from the FSAverage32k spherical mesh. Obtaining these outputs involves initial resampling of the FSAverage32k sphere to produce a lower resolution source model, typically an 8 k spherical mesh. Registration of the previous low-resolution sphere in the HCP spaces and re-meshing, a process that rebuilds the mesh topology, leads to the final 8 k meshes. These include the T1w FSAverage mesh, which is meant for the forward and inverse models in the subject individual space (Figure 3A top row). Unfortunately, a problem arises with the distortions in the individual geometry due to resampling and remeshing. We illustrate geometrical distortions with the first- and second-order surface metrics (Figure 3A) that are compared with similar metrics due to the resampling and remeshing directly in the individual space (Figure 3B).

We acknowledge that preserving geometry or these metrics might not be essential for some forward or inverse models. However, they are essential for CiftiStorm for processing EEG head models and lead fields involving the brain, cerebrospinal fluid conductivity compartments, and inverse solutions involving geometrical information (Figure 3B) such as the graph Laplacian prior (Figure 3B top row) (Nunez et al., 1994), depth compensation weighted in curvature (Figure 3B middle row) (Lin et al., 2006), or normal rotational invariance prior (Figure 3B bottom row) (Haufe et al., 2008). Consequently, our strategy for the source processing module is illustrated in Figure 3C. Procedures implementing this strategy are included in the following MATLAB modules fully developed along with CiftiStorm:

- fwd_source_model.m performs 8 k resampling and remeshing in the T1w FSAverage32k space for the source model of each individual. These procedures are carried out by a CiftiStorm module leveraging the standard MATLAB computational geometry libraries, which are also commonly applied within FieldTrip and Brainstorm registration pipelines for other purposes.
- fwd_interpolant.m builds the interpolant of the inverse solution or other ESI metrics between the T1w 8 k mesh and the T1w FSAverage32k mesh. This interpolation, a CiftiStorm built-in MATLAB module, is a 32kX8k matrix representing the weighted average operator with weights defined as the surface-based geodesic distance. This matrix is defined once for the individual. It is used to project any metric, such as vectors representing the instances of an inverse solution or a matrix representing their second-order statistics.

#### 2.2.2 Head model and lead field

The fundamental problem behind the EEG lead field calculation (valid also for MEG) is to obtain the electric potential produced in the head by each brain source, represented as a unitary dipolar current element (Turovets et al., 2008; Jochmann et al., 2011; Dannhauer et al., 2012; Azizollahi et al., 2016; Vorwerk et al., 2018). We adhere to standard assumptions about the head medium, considering it to be passive (non-magnetic) and characterized by broadband stationarity at all frequencies within the spectra of neural dynamics. This assumption leads to the quasistatic regime of electromagnetism. In this context, the central problem is the solution to the equation governing the electric potential induced in the medium by a current source density (Geselowitz, 1967; Vladimirov, 1976). The specification of this equation depends on the individual source model, head model geometries, and conductivities. Obtaining this information necessitates having at least the T1w sMRI for the individual (Akalin Acar and Makeig, 2013).

Obtaining the electric potential, which depends significantly on the type of head model, can become a computationally intensive and error-prone process. There is a tradeoff for head models that balances simplicity vs. detail and numerical accuracy/efficiency vs. realism in the subsequent lead field calculations. Below, we define the types of head models and lead fields CiftiStorm considers.

- The most straightforward head model is the homogeneous one (Nunez, 1974; Nunez et al., 1994; Jirsa and Haken, 1997). This idealization assumes equal conductivities for the brain tissue, head tissues, and air, substituting a mean field corrected conductivity for the actual ones. The essential advantage of this type of model is the generation of a smooth (analytic) and artifact-free lead field, calculated efficiently with explicit formulas for each geometry. It is important to note that the homogeneous lead field formula represents the boundary-free solution of the Poisson equation. This formula is, in turn, a type of baseline solution, the algebraic homogeneous term affecting the realistic lead field (with heterogenous conductivities), that must be obtained by numerical integration.
- The head model used for Electrophysiology Source Imaging (ESI) is designed with a moderate level of detail and is widely accepted for its approach. It bases its calculations on the assumption that the conductivities of brain tissue and other head components—like cerebrospinal fluid, skull, and scalp—are uniform and directionally uniform (isotropic) (Hamalainen and Sarvas, 1989; Hämäläinen et al., 1993; Riera and Fuentes, 1998). To solve the related equations, this model uses the Boundary Elements Method (BEM) to numerically address the Poisson equation—a specific type of partial differential equation—in a simplified (weak) integral form (Fuchs et al., 2002). The accuracy of these numerical solutions heavily depends on the head’s geometric properties being regular and smooth, which is encapsulated in what is known as the head model matrix. This matrix defines the electric potential in the head, which is influenced complexly by the positions of brain sources and virtual sources near the boundaries between different types of tissues. The calculation benefits from using these virtual sources—a mathematical strategy that simplifies the weak integral formulation—since their effects are directly proportional to the differences in electric potential and conductivity at each source point (Kybic et al., 2005). This approach allows for a more manageable calculation of electrical activity within the brain, considering the complex interplay between brain source locations and the electrical properties of head tissues.
- As mentioned, a template head model is widely used, assuming moderately detailed brain tissue and head tissue piecewise homogeneous and isotropic conductivities (as in II above). The template can also consider heterogeneous and anisotropic conductivities due to information. Thus, head models and source models from template T1w sMRIs such as MNI-ICBM152 (Fonov et al., 2011) or MNI-AutoReg (Collins et al., 1994) are most common for ESI applications when the individual sMRI acquisitions are not available or usable or when avoiding the intensive computational cost and errors of individualized lead field calculations. While such a template or a homogenous head model may suffice when limited ESI resolution is acceptable, this approach may not be suitable for higher-resolution inverse solutions based on high-density MEG or EEG recordings.

**Figure 4:**
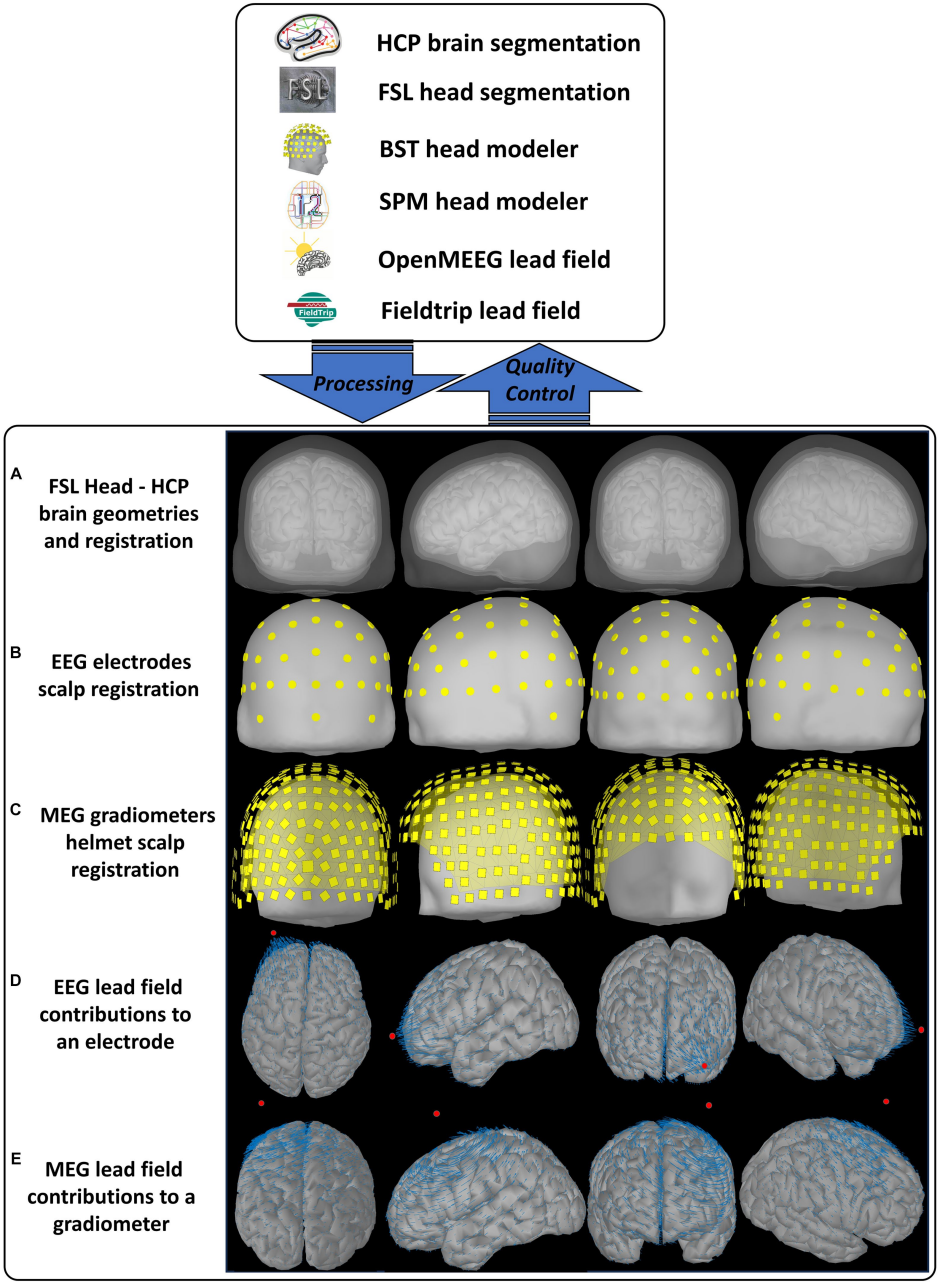
CiftiStorm head model, lead field processing, and quality control workflows. Top box: The neuroinformatic tools included in our pipeline and their different roles. Bottom box: The primary outputs are later targeted by the manual or automatic quality control analysis. An illustration aims to show the expected outputs under ideal working conditions, obtained from the high-quality MNI-ICBM152 image and its legacy structural processing (Sec. 2.1.2) and source model processing (Sec. 2.2.1). Outputs are **(A)** the head model comprising four structures: cortex, inner skull, outer skull, and scalp surfaces. These surfaces are due to the HCP brain and FSL head processing, postprocessing, and registration via the Brainstorm head modeler utility. **(B)** The registration for EEG electrodes and their layout with the scalp surface. **(C)** The registration with the scalp surface for MEG magnetometers and their helmet layout. For optimal sensor registration in **(B)** and **(C)**, the CiftiStorm pipeline leverages utilities from both the Brainstorm and the SPM head modelers. **(D)** The lead field for an EEG electrode. **(E)** The lead field for a MEG magnetometer. We use the reciprocal representation of electromagnetism to illustrate at the source space (cortex) the distinct vector field regimens for EEG and MEG sensors. Both are due to BEM computations employing the Brainstorm OpenMEEG or alternative FieldTrip utilities.

The CiftiStorm head model and lead field processing modules are implemented based on the Brainstorm suite and produce standard directory and file formats. Our design of these modules outlined below is tailored to targeting critical quality factors integrating functionalities from diverse neuroinformatic tools. Figure 4 illustrates the MNI-ICBM152 T1w sMRI template outputs, the processing, and quality control workflows. These are implemented in the following MATLAB modules:

- fwd_head_model.m: First, the non-brain tissue employing FSL BET and skull-stripping is extracted and refined using a series of FSL stat and math commands. The non-brain tissues (scalp, skull, and cerebrospinal fluid) are extracted as tetrahedral volume meshes. These meshes are linearly realigned to the brain tissue identified during the structural pipeline processing stage via the FSL FLIRT utility and post-processed via the Brainstorm head modeler utility to outline their boundaries, which define the inner skull, outer skull, and scalp triangular surface meshes. Additional regularization employing smoothing of the second-order surface properties, such as triangle size, normal, and curvature, leads to the final head model and promotes the subsequent numerical BEM integration stability. Sensor registration leverages a second utility from the SPM head modeler. An MNI-registered scalp template is then non-linearly wrapped to the individual’s actual scalp and employed as a reference scalp to optimize the registration of EEG sensors or the MEG helmet with the head model. Then, the sensors are projected to the actual scalp in the case of EEG or co-registered with the helmet in the case of MEG.
- fwd_lead_field.m: Using the processed head model and registered sensor files, lead fields are calculated via the BEM method from Brainstorm that invokes OpenMEEG software included as a Brainstorm plugin. It is also possible to use other BEM implementations from FieldTrip that provide similar results. Calculations follow the Brainstorm recommendations (defaults) to tune the essential conductivity and geometrical BEM parameters. The same head model and sensors are then used to compute a second lead field and baseline for quality control via the homogenous method. When no individual head model is available, the BEM method is calculated only once for the whole dataset, after a minimal registration step between the sensor template (specific to each dataset) and the head model template.
- fwd_control_loop.m: Quality control and corrections initiate creating an interactive graphic report of critical geometrical and numerical indicators for the head model and lead field quality. A report can be generated at any processing stage by invoking this module, which checks the available head model or lead field files. Within a loop between the visual report inspection and manual or automatic reprocessing, cases can be flagged for further consideration and corrected for altered indicators, calling back the modules fwd_head_model.m and fwd_lead_field.m.

**Figure 5:**
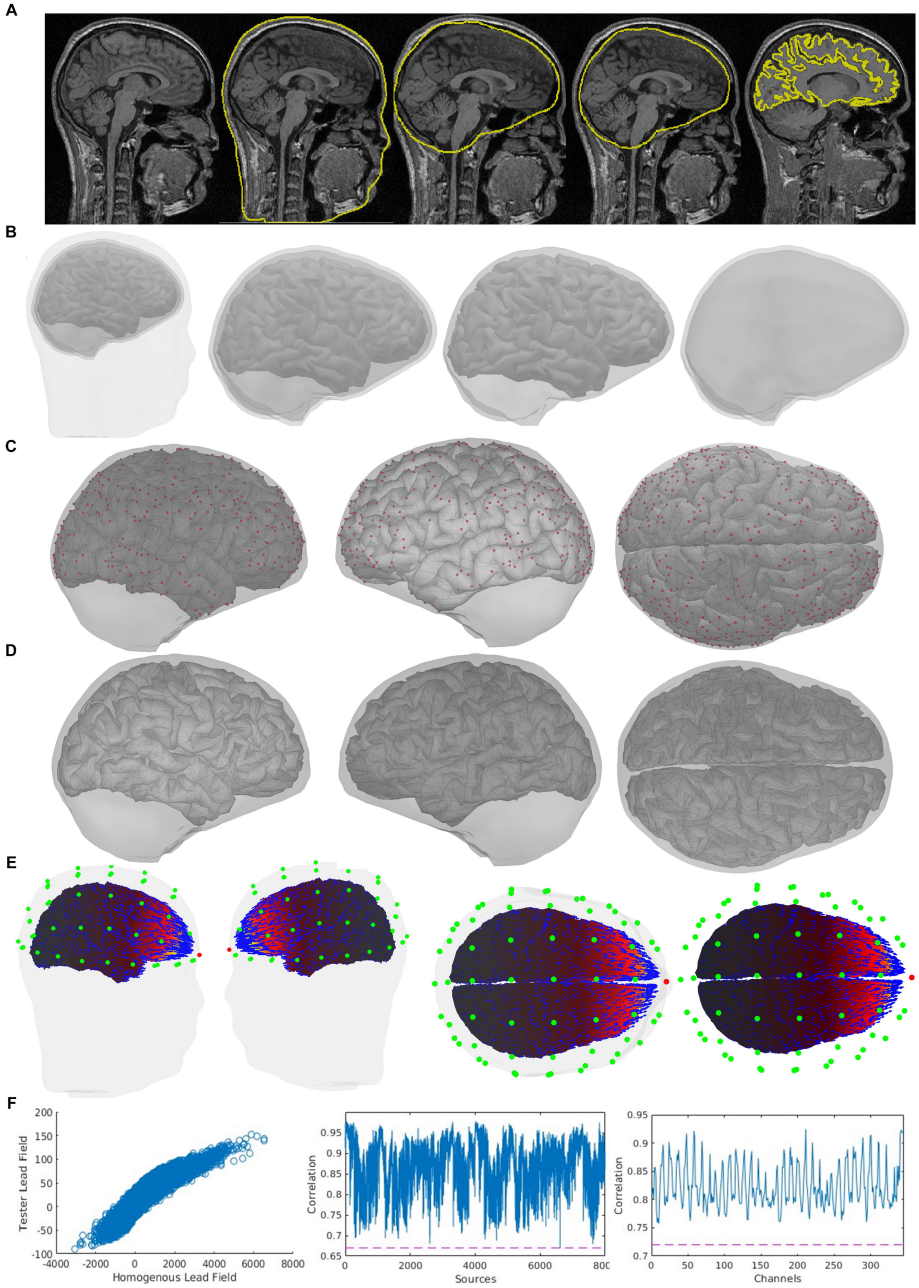
Quality control indicator information of the same case is shown in Figure 4 in an expanded way, which is also included in CiftiStorm’s graphic interface and printed reports. These indicators include **(A)** the sMRI and the registration of source and head models with it (from left to right: the scalp, outer skull, inner skull, and brain surfaces); **(B)** mutual registration between the head model and the source model, from left to right, first one: the scalp, outer skull, inner skull, and brain surfaces, second one: inner skull, outer skull and brain cortex, third one: inner skull and brain cortex, last one outer skull and inner skull; **(C)** left, right and top view of the inner skull and brain cortes registration, in red color: the points situated too close between them; **(D)** inner skull and brain cortex registration after distance correction process; **(E)** the vector field distribution (blue arrows) for a user selected sensor (red dot), sensors (green dots); **(F)** correlation tests between the tested lead field and the homogeneous lead field and their corresponding Pearson correlation coefficient, first one: tested and homogeneous lead field correlation, second one: sensor-wise correlation between tested and homogeneous lead field, and last on: source-wise correlation between tested and homogeneous lead field.

Figure 5 offers the detailed indicator information summarized in Figure 4 and considered by the quality control loop “fwd_control_loop.m. These indicators include (a) the typical 2D views (slices) of the sMRI and the source and head model-sMRI registration; (b) the 3D views of the head model, including the source model, inner skull, outer skull, scalp surface, and their mutual registration; (c) for a source model surface, the points that are situated too close to the inner skull surface; (d) the corrected inner skull surface under a user selected optimal surface-surface distance criterion; (e) the vector field distribution for a user selected sensor; (f) the 2D linear plots representing goodness-of-fit between the BEM lead field and the homogeneous lead field and their corresponding Pearson correlation coefficient, which must be above a user-selected correlation threshold. Two types of correlation coefficients are implemented here, the sensor-wide (for each source) and the source-wide (for each sensor), as described in our previous study (Riaz et al., 2023). Alterations in this indicator are sensitive to the numerical instability of the BEM methods originating from critical surface–surface distances and non-smooth local surface irregularities in the head model.

Several options are implemented to correct head model/lead field artifacts:

- The sensor layout registration automatically includes the default Brainstorm and CiftiStorm automatic corrections. However, further corrections can be carried out manually in case of severe artifacts, guided by visual inspection via the CiftiStorm graphic interface.
- For minor artifacts of the inner skull, outer skull, and scalp geometrical and their registration, automatic corrections may be implemented using the MNI-registered template non-linearly registered to the individual geometry using the Brainstorm or SPM head model utilities.
- Flagged surface–surface distance artifacts (below a threshold) are automatically corrected by warping the internal surface at that point to increase the distance. Subsequently, the quality control procedure is repeated.
- Finally, in case of severe artifacts due to a failed HCP or FSL segmentation or a non-existent at least a T1w sMRI, we recommend using a structural brainstorm template to obtain the lead field using a predefined source and head model geometry. This step requires sensor registration.

All these corrections require reprocessing by the “fwd_head_model.m” and the “fwd_lead_field.m” modules that are controlled by the “fwd_control_loop.m” module.

### 2.3 CiftiStorm inverse model pipeline

#### 2.3.1 Inverse solutions

CiftiStorm implements classical and recent ESI inverse solutions. We emphasize that special attention is given to the type of inverse solutions of cross-spectral tensors as a frequency domain measure of functional connectivity. However, quasilinear inverse operators also express these solutions, which allow the calculation of the usual source time series in the time or frequency domain.

The two classical inverse solutions included in our pipeline are the Beamformer Linearly Constrained Minimum Variance (LCMV) (Van Veen et al., 1997) and the Exact Low-Resolution Electromagnetic Tomographic Analysis (eLORETA) (Pascual-Marqui et al., 1994, 2006). The LCMV approximates an ideal filter to enhance activity at a given source and suppress the interference of others. eLORETA, by contrast, is designed to explicitly minimize localization errors of the maximum activity.

**Figure 6:**
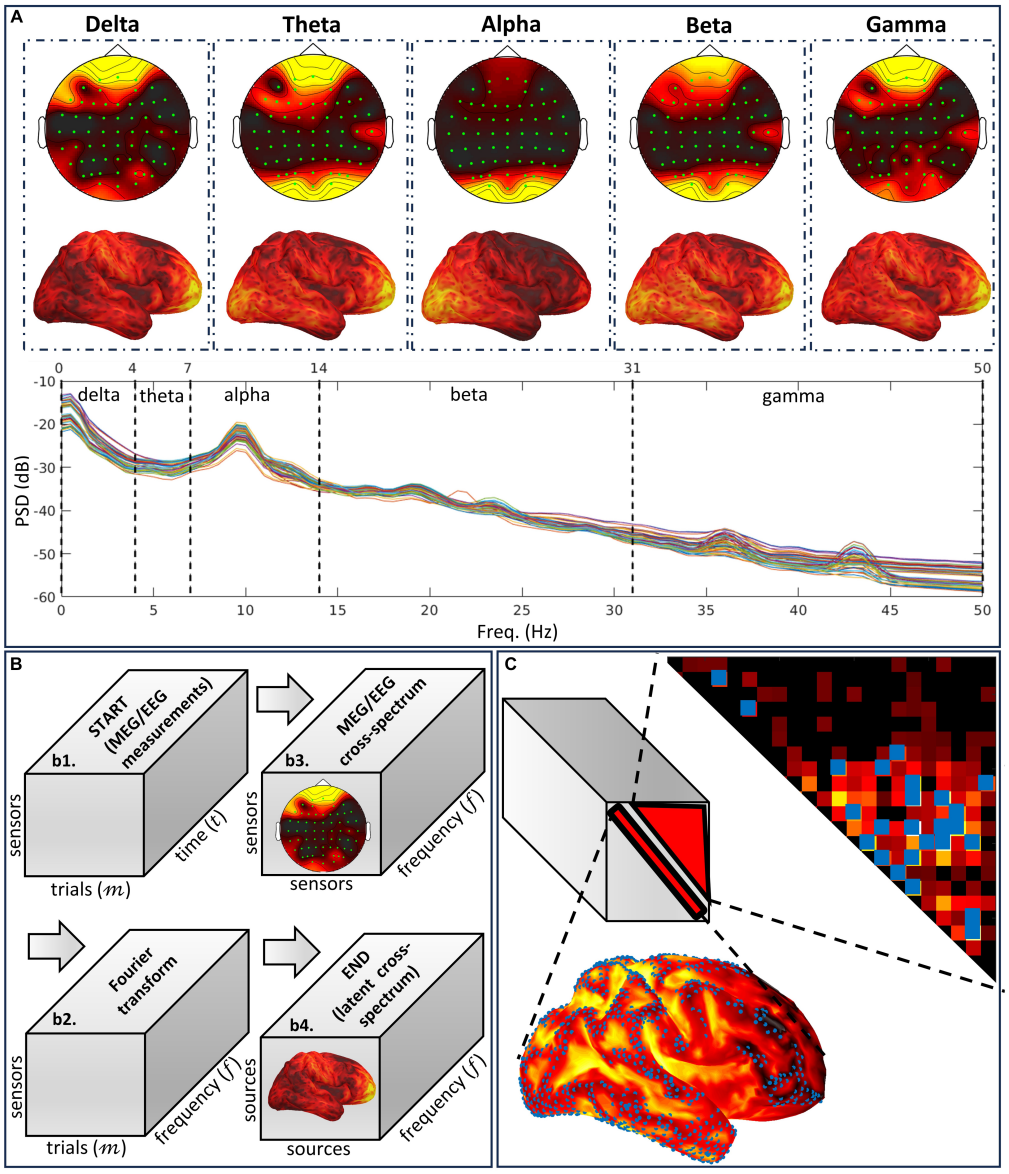
Illustration of the different notions embodying the cross-spectral methodology VARETA. **(A)** Characteristic spectral sensor topographies and cortical topographies composing the human resting state EEG spectrum. The cortical topographies that are determined by a conventional or cross-spectral inverse solution are the brain signature for band-limited oscillatory networks and the mechanism for activity associated with particular cognition and behavior. **(B)** Cross-spectral analyses of EEG sensor data and their sources involved in the cross-spectral inverse solution device, which follows the Fourier transform path. We describe this path as a series of tensor variables. The targeted variable is the theoretical source cross-spectral tensor, which must be determined by different methods from its sampled equivalent calculated for the data or the sources. **(C)** Regularization modes to resolve the high spatial ill-condition and dimensionality in the tensor target. These modes target independent distortions, measured as type-one and type-two leakage corresponding to the spectra and cross-spectra.

However, as we have recently shown (Paz-Linares et al., 2023a,b), these traditional inverse solutions for ESI are optimized to estimate activity and produce distorted cross-spectral estimators. CiftiStorm integrates novel cross-spectral inverse solutions optimized to estimate the source cross-spectral tensor within the theoretical framework of variable resolution electromagnetic tomographic analysis (VARETA) (Valdes-Sosa et al., 2000; Bosch-Bayard et al., 2001). We now briefly recap the VARETA notions to understand how to use CiftiStorm cross-spectral inverse solutions (Figure 6):

- The complete MEG/EEG inverse problem is formulated in the frequency domain, where time domain data and current source density are transformed using the Fourier or Hilbert transform. Under stationarity and mixing conditions in the frequency domain, asymptotic complex-valued Gaussian probabilities (Brillinger, 1983) apply to the Fourier or Hilbert transform of the current source density and the data. These probabilities fully specify second-order properties for oscillatory brain networks (Figure 6A), which produce MEG/EEG signals across the spectrum, including the well-known delta, theta, alpha, beta, and gamma activities, each with specific functional roles (Engel et al., 2001; Varela et al., 2001).
- VARETA is based on a Bayesian inverse problem formalism, defined by the following: probabilities of the data (likelihood), current source density (prior), and source cross-spectral matrix (hyperprior). It is, therefore, a three-level conditional model at each frequency (Bosch-Bayard et al., 2001). At each frequency, the source cross-spectral matrix (frontal slices of the tensor) specifies this model’s probabilities and inverse solution. The cross-spectral inverse solution (Figure 6B), a Bayesian estimator, maximizes a Bayesian cost function defined as −log posterior probabilities of the three-level conditional model.
- CiftiStorm provides two types of mathematical priors (Figure 6C) to regularize cross-spectral estimates (Valdes-Sosa et al., 2000).

1. Type-one regularization penalizes the diagonal entries of cross-spectral matrices (the spectra) or activation, thus controlling localization error and leakage distortion in these estimates.
2. Type-two regularization penalizes off-diagonal entries (cross-spectra), thus controlling localization error and leakage of the connectivity estimates (Gonzalez-Moreira et al., 2018; Palva et al., 2018).

Following the explanation given above, it is evident that applying the VARETA methodology to obtain cross-spectral inverse solutions is an intricate procedure (Bosch-Bayard et al., 2020). In what follows, we describe the specific CiftiStorm VARETA implementation in three MATLAB pipeline modules:

- inv_spectral_meeg_analysis.m initializes with minimal data preprocessing employing the EEGLAB ICA and ASR utilities in default mode. Preprocessed outputs are then imported and converted automatically to the Brainstorm utility. Processing continues obtaining the spectral metrics of MEG/EEG data via the Fast Fourier Transform (FFT) or Gaussian Filtered Hilbert Transform (GF-HT) algorithm, which approximates the FFT when the Filter bandwidth goes to zero. GF-HT is the preferred spectral transform when a more significant sample number, which equals the length of the MEG/EEG signal time series, is desired. The cross-spectral tensor of the MEG/EEG data is then computed as Hermitian covariance matrix of the FFT or GF-HT samples at each frequency. Since processing the full cross-spectral tensor is an intensive and, in many cases, unnecessary practice, the default mode uses band-filtered Hilbert transform (BF-HT). It computes a five-slice tensor representing the cross-spectra for the delta (0.5–4 Hz), theta (4–8 Hz), alpha (8–13 Hz), beta (14–30 Hz), and gamma (>30 Hz) bands.
- inv_sssbl_analysis.m: This cross-spectral inverse solution employs type-one regularization via the Spectral Structured Sparse Bayesian Learning (SSSBL) model (Paz-Linares et al., 2023a). Type-one regularization (described below in Section 2.3.2) is applied to resolve spatial distortions (type-one leakage, type-one localization error) caused by degeneracy (ill-condition) in the diagonal values (spectrum) of a matrix (cross-spectrum). The SSSBL model leads to a robust and scalable inverse solution even under the high ill-condition and dimensionally and the resulting distortions of an ESI situation with 19 electrodes (10–20) EEG system against a 32 k (FSAverage) source model. The SSSBL model outputs a tensor quasilinear inverse operator with frontal slices representing an iterated solver to the non-linear cost function optimization problem and the inverse solution at each frequency (or each frequency band). Tensor slices can be assumed as lead field pseudoinverse matrices, with a non-linear matrix function at each frequency with the argument on the data cross-spectral matrix. Thus, taking any data metrics (FFT, GF-HT, BF-HT, and cross-spectra) under this operator produces the analogous source metric. This operator is a compression method to avoid storing the source metrics while keeping only the data metrics. An additional output is the sparse support of sources at each frequency obtained by thresholding the spectrum elements significantly different from zero according to the SSSBL posterior probability.
- inv_higgs_analysis.m: Cross-spectral inverse solution that employs type-two regularization via the Hidden Gaussian Graphical Spectral model (HIGGS) (Paz-Linares et al., 2023b). Type-two regularization (described in Section 2.3.2) is applied to resolve spatial distortions in the Cartesian space product (type-two leakage, and type-two localization error) caused by degeneration (ill-condition) in the off-diagonal values of a matrix (cross-spectrum). HIGGS is an unbiased sparse inverse solution to obtain the cross-spectral precision tensor or precision matrices (frontal slices of the tensor) describing the oscillatory brain networks at each frequency as Hermitian graphs. Hermitian graph elements generalize the directed and undirected connectivity features encoded as amplitude and phase information across frequencies. The HIGGS output can be obtained with two types of methods: the two-step way, which inputs the source cross-spectral tensor initially determined by SSSBL to produce the corresponding cross-spectral precision tensor; the one-step way, which inputs the data cross-spectral tensor to estimate the source cross-spectral precision tensor and a tensor quasilinear inverse operator. These outputs can be used to obtain the additional metrics (FFT, GF-HT, BF-HT, and cross-spectra). Either method is based on the sparse support provided by the SSSBL statistics. A further provision is to impose sparse support for the graph elements at each frequency by retaining only significant cross spectra according to the HIGGS posterior probability.

Finally, we emphasize that a particular interest in frequency domain analyses, such as spectral factorization (Jafarian and McWhirter, 2012), phase–amplitude, or cross-frequency coupling (Sotero, 2016), may require additional considerations on the sparse support of the cross-spectral tensors. The cross-spectral inverse solution that only centers on individual frequencies or bands of frequencies is standard but may yield a discontinuous pattern of non-zeroed amplitudes across frequencies. Within the CiftiStorm, SSSBL, and HIGGS modules, we can apply joint statistical scores across the frequency domain to produce a transverse non-zeroed amplitude pattern.

#### 2.3.2 Integrated priors

Any combination of physical–mathematical priors currently integrated into the CiftiStorm inverse model processing pipeline can be applied to an inverse solution. Employing these priors may depend on user expectations around a particular geometrical, spectral, or mathematical source feature or even computational time and cost. Here, we provide the necessary background to guide the prior choice. However, an empirical demonstration of the incremental positive effect of incorporating geometrical priors in inverse solutions is provided in the following SSSBL study (Gonzalez-Moreira et al., 2020).

- The rotationally invariant prior. The cross-spectra are specified as a 3 × 3 complex-valued matrix of each source pair due to their origin in a 3D tensor field that defines current source density amplitude and orientation at cortical locations. This extra number of variables adds to the natural dimensionality and ill-condition in tackling a cross-spectral inverse solution. Considering a fixed field orientation by projecting the lead field in the normal direction of the cortical surface might be too restrictive and inaccurate (Haufe et al., 2008). We opt for a more relaxed condition, considering the probabilities of a vector field invariant under rotations around the surface’s normal direction. A rotationally invariant prior, described as spherical coordinates, assigns probabilities that decrease with the sagittal angle between the vector and the normal direction and remain invariant under the azimuthal angle of the vectors around the normal direction. This option is implemented via the standardization method, where transformations are applied to the design matrix in a multivariate regression problem. Then, the inverse transformation (de-standardization) is applied to the regression coefficient. This method first standardizes the lead field (design matrix), equivalent to projecting the lead field and obtaining the cross-spectral inverse solution restricted to the normal direction, a complex-valued scalar field for each source pair. Second, the inverse of the previous projection is applied to the inverse solution, retrieving the 3 × 3 cross-spectral matrix, a complex-valued tensor field for each source pair. Third, we obtain the maximum amplitude direction for each source from the 3D spectra, a real-valued and positive 3D vector field corresponding to pairs of the same source. Finally, the cross-spectral inverse solution is projected in the direction of maximum amplitude and again reduced to a complex-valued scalar. A logical parameter “field” is used in the SSSBL and HIGGS modules to control this prior.
- The surface curvature depth compensation prior. The bias of the inverse solutions is produced by surface curvature depth at every point of the cortical surface. We employ the definition of mean curvature (Van Essen et al., 2019), which is the average of the two principal curvatures for an inscribed ellipsoid at every surface point. The depth bias of inverse solutions regarding the observability of the sources’ distance from sensors has also been studied elsewhere (Lin et al., 2006). Here, we only refer to cortical curvature depth bias. In other words, the observability of the sources is higher around the gyri and lower around the sulci. This effect also varies in proportion to the curvature value at the source location (gyri or sulci). We implement a depth bias compensator based on the cortical curvature weights that factor (standardize) each source lead field. These weights are linearly transformed curvature values. Compensation is achieved by this linear transformation with different values for the gyri and the sulci of its slope and intercept, which we optimized in simulations. A logical parameter “curvature” is used in the SSSBL and HIGGS modules to control this prior.
- The graph Laplacian smooth prior. Lateral connections link the neighbor cortical sources, reinforcing their coactivation. Inverse solutions can consider such links by employing a geometric connectivity mode denominated graph Laplacian (Nunez et al., 1994; Pascual-Marqui et al., 1994). Integrating this prior is via the lead field transformation (standardization) by the graph Laplacian pseudoinverse. However, a problem arises from biological imaging protocols due to the strong bias of this transformation, which would be much more accurate employing the deformed graph Laplacian (Morbidi, 2013). This deformation is in the second-order expansion, around the minimum singular value, which uniformly converges to the graph Laplacian when this value rounds zero. A logical parameter “laplacian” is used in the SSSBL and HIGGS modules to control this prior.
- The parcellation smooth prior. Functional specialization of the brain areas can also be considered a prior for particular regularization models (Yuan and Lin, 2006). This model type is built in the SSSBL method, incorporating an additional Bayesian level of variational parameters that regularize the source spectra. The group prior takes effect when a unique variational parameter exerts its regularization effect on the group of sources belonging to a brain area. These areas are defined within the cortical parcellation produced in the structural pipeline. A logical parameter, “parcellation,” to control this prior is only available for the SSSBL module.
- The regularization type-one prior. Built-in SSSBL module; this prior combines the cross-spectral matrix quasinorm (Wilansky, 2013) and nuclear norm (Fan 1951) to create a two-fold effect. The quasinorm trace square root operator applied to a matrix pursues regularizing distortions in the spatial distribution of the spectra. The nuclear norm, trace operator applied to a matrix, pursues regularizing ill-condition of the cross-spectra. Combined norms are also known to be caused by adaptive matrix regularization problems, such as the Elastic Net nuclear quasinorm (Sun and Zhang, 2012). An alternative pathway is available for users preferring regularization priors within methods such as eLORETA (Pascual-Marqui et al., 2006) or Beamformers (Van Veen et al., 1997) over SSSBL. A logical parameter “sssbl_method” controls this pathway choice in the SSSBL module.
- The regularization type-two prior. Built-in HIGGS module (Paz-Linares et al., 2023b); this prior could be implemented as the Hermitian Graphical Least Absolute Shrinkage and Selection Operator (HGLASSO), also vectorized p1-norm applied to off-diagonal entries of a matrix, or the Hermitian Graphical Ridge (HGRidge), also vectorized squared p2-norm or Frobenious norm applied to a matrix. Either norm is fundamental to producing an inverse solution and regularizing the ill-condition of the cross-spectral precision matrices. However, the HGLASSO norm is key to pursuing an unbiased sparse pattern of the precision matrix. The Frobenious norm is a non-sparse biased but analytical alternative to HGLASSO. A logical parameter “higgs_method” controls this choice in the HIGGS module.

## 3 Results

### 3.1 Geometrical artifact corrections

We carried out an initial test of concept for CiftiStorm in three public datasets: the Human Connectome Project (HCP) (Larson-Prior et al., 2013), the Cuban Human Brain Mapping Project (CHBMP) (Valdes-Sosa et al., 2021), and Healthy Brain Networks (HBN) (Alexander et al., 2017). The raw CiftiStorm output data are curated by the high-performance computer center of the Neuroinformatics Collaboratory at the University of Electronic Science and Technology of China (UESTC). The Supplementary Table S1 includes the description of the curated data and available imaging modalities. The same data are mirrored in the Compute Canada and the Cuban Neuroscience Center (CNEURO) as part of the CCC-AXIS work front (Evans et al., 2020).

The processing first obtains structural outputs by applying the standard (Sec.2.1.1) and legacy (Sec.2.1.1) CiftiStorm pipelines to raw HCP data and the legacy pipeline to raw CHBMP and HBN data. Subsequent processing obtains the source and head model outputs of all the data (HCP, CHBMP, HBN) by applying the CiftiStorm forward model pipeline. Artifact annotations to these outputs were created within the quality control loop as the result of extensive analysis by our team experts. These artifact annotations follow a standard questionnaire prepared following the HCP quality control recommendations (Marcus et al., 2013). We conducted a final trial on the annotated artifacts, calculating their scores using item response theory (Vega et al., 2021; Riaz et al., 2023).

We proceeded with the case exclusion or correction based on their specific annotations. Then, we investigated the similarity of the standard and legacy structural pipelines and the forward model pipeline (Sec.2.2) across all databases (HCP, CHBMP, and HBN). Sample data were created for the next inverse model pipeline (Sec.2.3), including quality case outputs such as the non-annotated or fully corrected ones.

**Figure 7:**
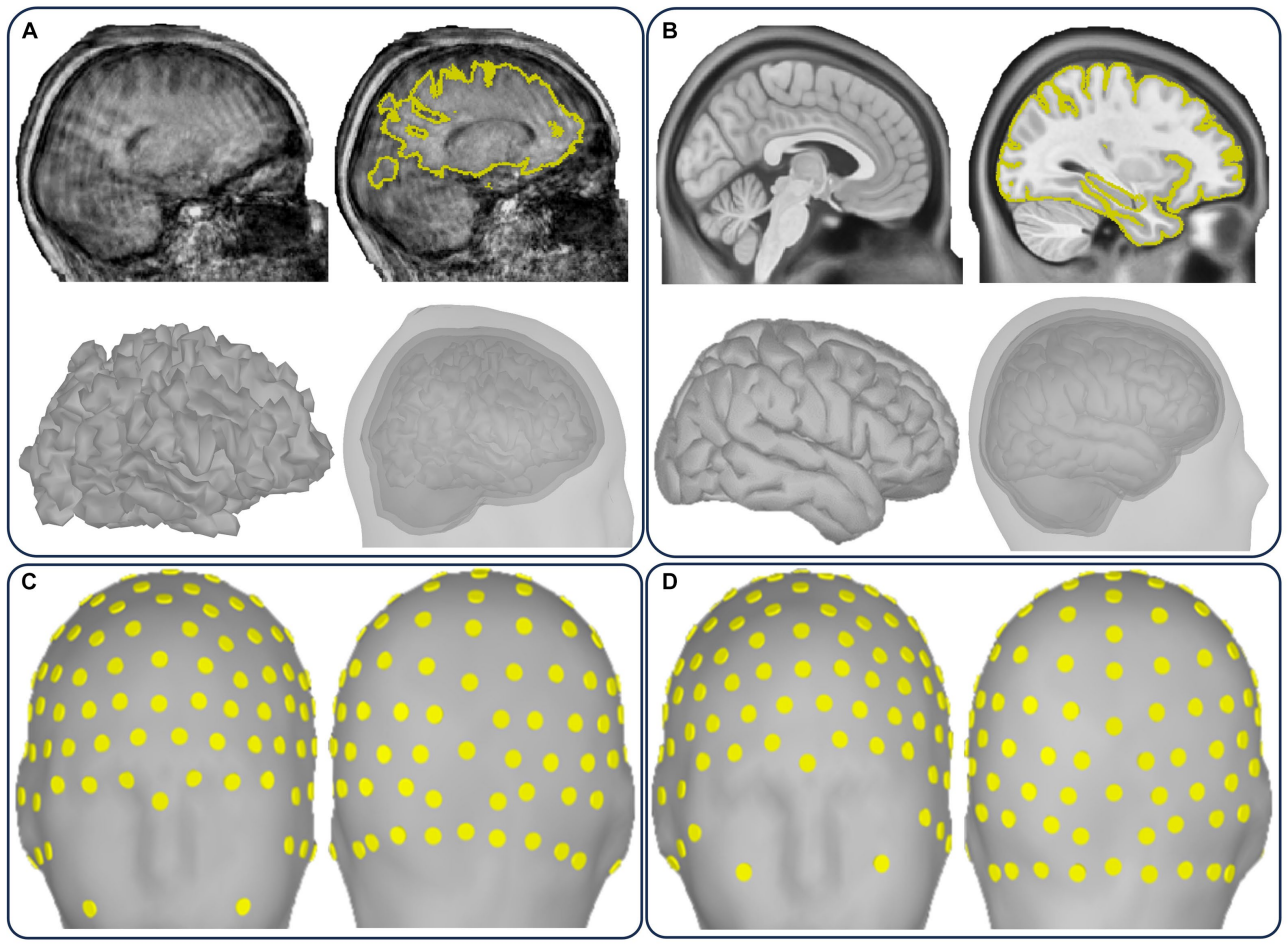
Case flagged as “artifactual” for two major geometrical artifacts in Figure 5 and their solution in the quality control loop. **(A)** Individual sMRI showing movement artifact and the corresponding artifactual source model and head model outputs. **(B)** ICBM-152 sMRI, source model, and head model template substituting the individual outputs. **(C)** Incorrect EEG layout. **(D)** Corrected EEG layout.

Figure 7 includes geometrical annotations for some key quality factors in Figure 5. We detect significant geometrical artifacts caused by movement or noise (Figure 7A) in both the T1w and T2w sMRI of the HBN and CHBMP datasets. sMRI noise appears in artifactual cases covering the head tissue and exterior areas, and movement appears as blurring in the head tissue areas. The decision to exclude or postprocess is quite challenging for such cases due to the cost/benefit relationship. Although postprocessing may work in cases by tunning the FreeSurfer or FSL parameters, doubtful brain or head segmentations can ultimately be solved by replacing the sMRI with a template (Figure 7B). The widespread issue of electrode alignment (layout) in EEG (Figure 7C) is solved in part manually and automatically refined later (Figure 7D). After geometrical corrections, the cases are looped back to structural and forward model processing to produce their new outputs.

### 3.2 Lead field artifact corrections

**Figure 8:**
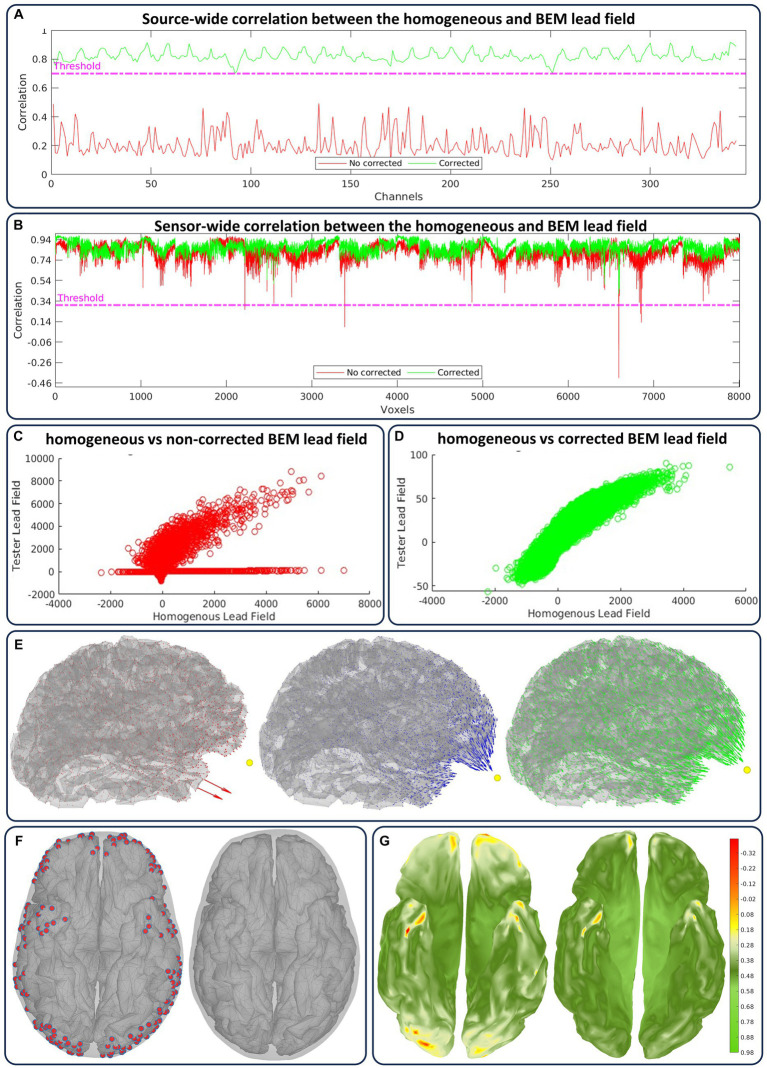
Case flagged as “artifactual” for the lead field artifacts in Figure 5 and their solution in the quality control loop. We show the minimum correlation value between the homogeneous and actual lead field outputs before (red) and after (green) correction in **(A)** across sources (for each sensor) and in **(B)** across sensors (for each source). The linear fit of both lead fields relative to the same correction is shown in **(C)** before and **(D)** after. **(E)** the artifactual, homogeneous, and corrected lead fields are shown from left to right. **(F)** From left to right, and under distance criteria, the sources labeled (red) as too close to the inner skull surface and the sources after correction. **(G)** From left to right, there is a cortical colormap of the source correlations before and after correction.

Geometrical annotations also reflect the lead field quality and its key factors, which are part of the quality control loop and the artifacts annotation processes. We emphasize that the geometry of the source model (Sec.2.2.1), head model, and lead fields (Sec.2.2.2) have a multifactorial relationship that a closed set of parameters cannot condense. However, we consider the relative surface–surface distance within the head model, including the source model, which critically influences the lead field’s numerical accuracy. Figure 8 includes the type of lead field artifact annotations and their treatment in the quality control loop.

We consider two correlation modes between the homogenous and actual lead field outputs: for a sensor (represented by a lead field row), the source-wide correlation, and for a source (represented by a lead field column), the sensor-wide correlation. The automatic quality control of the lead field is applied sensor-wise (Figure 8A) and source-wise (Figure 8B), simultaneously overseeing the two correlation modes (Riaz et al., 2023). We also manually explore the linear fit between both lead fields, which reveals more detailed information regarding the correlations (before correction Figure 8C and after correction Figure 8D). Indeed, correlations and linear fit are sensitive to the type of non-smooth or singularity lead field artifacts that appear for several sources and reflects in sensor correlations. Cortical maps highlight these artifacts and their correction in terms of the field amplitude and orientation (Figure 8E).

The corrections are applied by forcing the inner-skull local triangle mesh outwards till a minimum distance to the target source is achieved (Figure 8F). This case was flagged as “artifactual,” and corrections were applied due to detected correlations below 0.7 for sensors and below 0.33 for sources. We have predefined the minimum distance value and investigated its effect across the present datasets, but this value can be updated iteratively within the CiftiStorm quality control loop. After correction, the source correlation values are updated (Figure 8G), always exhibiting their direct relationship to local distance manipulation or lead field artifacts.

We emphasize that artifact annotation and correction are critical for the automatic CiftiStorm quality control for the lead field. We can thus detect many of the geometrical artifacts delivered within structural and forward model processing. A previous study (Riaz et al., 2023) reveals sensitivity to detecting electrode layout deformations, non-smooth surfaces, sMRI artifacts, and failed FreeSurfer or FSL segmentations.

### 3.3 Comparing EEG/MEG inverse solutions

We study the similarities and differences of CiftiStorm inverse solutions (Sec.2.3.1) across the CHBMP EEG and the HCP MEG datasets (Riaz, 2021). The inverse solution module under analysis was SSSBL, which incorporates the type-one regularization priors as well as the geometrical priors (Sec.2.3.2). Our working hypothesis is “for the same condition (resting state) the spatial distribution of source spectra estimated by inverse solutions across all frequencies of MEG and EEG are equivalent” (Riaz et al., 2020). For this study, a sample data of 45 cases (participants) was created from the CHBMP dataset, and another sample data of 45 cases from the HCP dataset yielded a balanced sample size. We selected the cases by filtering them according to their scores in the geometrical and lead field artifact annotation analysis and their age between 22 and 35 years.

**Figure 9:**
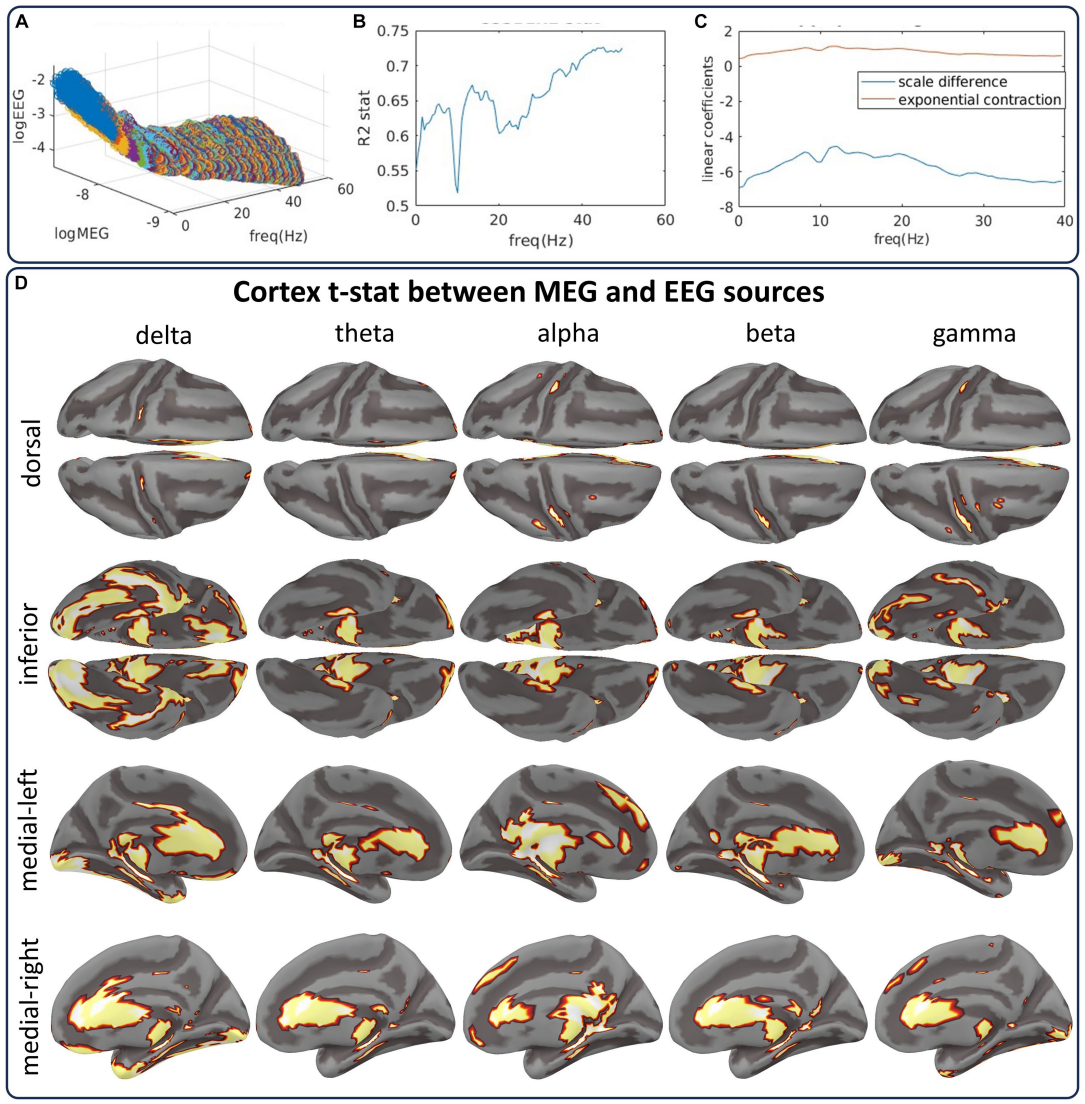
The CiftiStorm inverse solution pipelines from the CHBMP EEG and HCP MEG datasets show differences between their source spectra. **(A)** Scattergram manifold illustrating the global differences between MEG and EEG source log spectra. The large differences are due to the properties of either type of acquisition or preprocessing style. **(B)** Results of the regression using a linear model that represents these differences as a scale and a slope parameter. **(C)** Goodness of fit for this linear model. **(D)** Random permutation test outcomes of the differences for delta, theta, alpha, beta, and gamma-low bands.

The study initiates with spectral processing of the sample data, employing a narrow-band Gaussian Filtered Hilbert Transform (GF-HT) for each frequency between 0 and 50 Hz, with a resolution of 0.5 Hz and a filter bandwidth of 1 Hz. Subsequent processing is via the SSSBL inverse solution. Figure 9 includes the analysis of source spectra and statistical tests to explore our hypothesis for CHBMP EEG and HCP MEG acquisitions in the resting state condition. Before any statistical comparison, the log-spectra transformation and linear regression were applied considering a model of the global differences between the MEG and EEG source spectra:


$$\text{Model}:\text{MEG}\left(s,f,c\right)=e^{a\left(f\right)}\text{EEG}\;{\left(s,f,c\right)}^{b\left(f\right)}$$

$$\text{Log-spectra}:\text{MEG}\leftarrow \log\left(\text{MEG}\right)\;\text{and}\;EEG\leftarrow \log\left(\text{EEG}\right)$$

$$\text{Regression}:\text{MEG}\left(s,f,c\right)=a\left(f\right)+b\left(f\right)\text{EEG}\left(s,f,c\right)+E\left(s,f,c\right)$$

In this model, “MEG” and “EEG” are 3D tensors representing the spectra of MEG and EEG sources. The tensors comprise 64 k sources (denoted with s), 100 frequencies (denoted with f), and 45 cases (denoted with c).

The manifold in Figure 9A represents the mean log spectra of MEG and EEG source pairs at each frequency scattergram. The manifold highlights two types of differences encoded by the regression parameters $a\left(f\right)$ and $b\left(f\right)$:$a\left(f\right)$ (intercept) is the frequency-dependent MEG and EEG spectral power difference induced by acquisition and preprocessing methods. We model this difference as a scale parameter of the source spectra reported in previous simultaneous MEG/EEG studies (Dehghani et al., 2010). Figure 9C (blue curve) shows this parameter’s strong frequency-dependent behavior.

$b\left(f\right)$ (slope) is the difference in regimes of spatial decay regarding the distance between sensors and sources of MEG and EEG. We model this difference as an exponential contraction parameter of the EEG source spectra reported in previous studies on the MEG/EEG sensitiveness to sources (Piastra et al., 2020). Figure 9C (red curve) shows the stable behavior of this parameter across frequency.

$E\left(s,f,c\right)$ (reminders) represents the reminders or residuals of the Regression: Model:. Reminders may comprise MEG/EEG noise, different preprocessing, or interindividual variability.

The $R^{2}$ coefficient of the Regression: Model: in Figure 9B shows the goodness-of-fit between MEG and EEG source log spectra across all frequencies. We proceed after regression, studying the source differences with a random permutation test to the spectra distribution. Figure 9D summarizes the test outcomes for five bands (delta, theta, alpha, beta, and gamma-low) in cortical topographies. The cortical topographies are for a set indicator function of the binary outcomes. The set summarizes any source showing a significant statistical difference at any frequency within the band. This test yields striking similarities between the EEG and MEG source spectra and only a few differences associated with EEG’s low sensitivity to subcortical and interhemispheric sources. Differences are due to a significance level of 0.01. This level was applied to determine thresholds for the distribution of the test maximum absolute value taken across all sources and frequencies.

## 4 Discussion

Developing high throughput pipelines for electrophysiological source imaging (ESI) has been the target of numerous efforts, e.g., megconnectome (Larson-Prior et al., 2013), Fieldtrip (Oostenveld et al., 2011), and Brainstorm suite (Tadel et al., 2011). However, the diversity in data acquisition technologies, protocols, standards, and structural, forward model, and inverse model pipelines are causing a massive quality gap in the ESI outputs across and within datasets. This gap results in overwhelming variability in the inverse solutions leveraged within ESI analyses, leading to issues with replicable, reproducible, or transferable ESI research in basic or clinical neurosciences and multimodal neuroimaging.

A prominent effort introducing ESI guidelines, standards, and pipelines such as the megconnectome toward a broader multimodal neuroimaging connectomics research framework was the Human Connectome Project (HCP) (Van Essen et al., 2012a,b, 2013; Glasser et al., 2013; Marcus et al., 2013). We introduced CiftiStorm, an HCP megconnectome pipeline compliant with the Brainstorm suite, to target the root causes of such ESI problems. Notably, CiftiStorm development was appointed by the Global Brain Consortium (GBC) (Valdes-Sosa et al., 2022), explicitly responding to the EEG ESI challenge.

As we corroborated for the CHBMP EEG and HCP MEG (Sec.3.3 and Figure 9), our pipeline could potentially lead to analogous ESI results across the human MEG/EEG spectrum. The statistical MEG/EEG comparison test provided central evidence of the CiftiStorm’s properties. We carried out this test under the following hypotheses: (a) the principal cause for MEG and EEG signals is the cortical current source density, and (b) cortical sources causing these signals must be approximately the same. In this sense, the test outcomes constitute more evidence than proof for the classical theories of the MEG/EEG origin (Nunez et al., 1994; Dehghani et al., 2010; Piastra et al., 2020; Riaz et al., 2020).

Broader consequences follow from this outcome, and our definition for tested MEG source variables is the Hilbert transform of narrow-band filtered current source density. These source variables are essential for transferable MEG and fMRI connectomics research since they provide the link between oscillatory networks underlying the MEG and principal components of the fMRI (Brookes et al., 2011a; Hipp et al., 2012; Hall et al., 2014; O’Neill et al., 2015; Tewarie et al., 2016). Thus, our results may also represent a striking incremental step toward EEG/fMRI fusion and broader EEG ESI integration within the multimodal neuroimaging framework.

Such an integrative neuroimaging perspective is in the guidelines identified by the GBC and similar international initiatives such as the HIBALL (Amunts et al., 2016), CCC-AXIS (Evans et al., 2020), CHBMP (Valdes-Sosa et al., 2021), UKB (Miller et al., 2016), and HBN (Alexander et al., 2017). Following the GBC guidelines, the CiftiStorm source code and graphic interface are open-source to help other groups replicate, reproduce, or transfer the results presented in this study.

It is essential to underscore that our pipeline is designed to enhance the use of novel MEG/EEG inverse solutions (Sec2.3 and Figure 6). They have their basis in the methodology of variable resolution electromagnetic tomographic analysis (VARETA) (Valdes-Sosa et al., 2000; Bosch-Bayard et al., 2001). As we demonstrated in a previous report (Riaz et al., 2020), such high levels of similarity is unreachable via existing inverse solutions such as the low-resolution electromagnetic tomographic analysis (LORETA) methods (Pascual-Marqui et al., 1994, 2006; Pascual-Marqui, 2002). Neither are they possible with Beamformer methods linearly/multiply constrained minimum variance (L/MCMV) (Van Veen et al., 1997; Piotrowski and Yamada, 2008).

As argued in Section 2.3, our results confirm the favorable regularization effect of mathematical priors integrated into Bayesian methods, such as our Spectral Structured Sparse Bayesian Learning (SSSBL) (Paz-Linares et al., 2023a) and Hidden Gaussian Graphical Spectral Model (HIGGS) (Paz-Linares et al., 2023b). Recent literature has also highlighted the power of Bayesian methods such as SSSBL for many applications, including MEG/EEG denoising (Hashemi et al., 2022), inverse solutions (Hashemi and Haufe, 2018), or statistical analysis (Wang et al., 2023). In addition, note that we incorporated morphometric information on the sources (Nunez et al., 1994; Pascual-Marqui et al., 1994; Lin et al., 2006; Yuan and Lin, 2006; Haufe et al., 2008) into SSSBL or HIGGS to incorporate as another important geometrical priors.

Our results are determined by the quality control loop applied to resolve the lead field artifacts (Sec.3.2). As we argue here, not only the inverse model but also the lead field accuracy is crucial in obtaining precise inverse solutions (Vorwerk et al., 2018; Piastra et al., 2020). Furthermore, the corrections applied within CiftiStorm could potentially increase the number of adequate quality cases for future studies employing other datasets. Fundamental to developing and calibrating our forward model (lead field) pipeline was the processing of a collection of 1,251 sMRIs via the structural pipeline (Sec.3.1). This collection was courtesy of the Healthy Brain Networks (HBN) initiative (Alexander et al., 2017). Our quality control loop identified 60 “artifactual” lead fields from artifact annotations in this calibration stage.

The CiftiStorm’s numerical accuracy index detects artifacts based on correlations, which our previous study introduced for the first time (Riaz et al., 2023). When the “artifactual” lead fields were compared to another 60 “acceptable,” we found three main reasons for them being declared as “artifactual. This finding is explained by the corresponding artifact corrections of the CiftiStorm pipeline. The first reason was the blur/noise of the sMRI causing inaccurate segmentation; the second was the imperfect sensor alignment, and the third was the critical surface-surface distance within the source and head models.

In case of an evident failed sMRI segmentation, an initial correction was retrying the segmentation after tunning FreeSurfer’s and FSL’s parameters. However, in cases where the sMRI still could not be adequately segmented, we flagged its outputs as “incorrigible. The “incorrigible” flagged sMRI outputs were then substituted by template outputs created by applying the legacy structural pipeline to an MNI-registered sMRI (Collins et al., 1994; Fonov et al., 2011). The use of templates is still common and leads to approximated ESI analysis from EEG and MEG (Litvak et al., 2011; Ashburner et al., 2014). Specific anatomy configuration files allow the switching of individualized or predetermined templates. This initial correction helped isolate the second and third types of lead field artifacts. The user must know the limitations of applying brain templates, which always entail a specific loss of localization accuracy. There is justified concern about the use of templates in ESI. However, one of the results of the Cuban Human Brain Mapping Project was the actual comparison of hundreds of ESI based on individualized head models with several types of templates (Valdés-Hernández et al., 2009). Although this study confirmed the higher accuracy of individualized templates, it was also found that the localization error incurred with different templates was acceptable for specific course-grained applications.

The second correction applied was the EEG (MEG) sensor registration. This correction was done via an automated and manual utility within the CiftiStorm’s quality control loop. The automated correction replaced the MNI-registered scalp to improve the sensor alignment in the actual scalp. The manual correction was in the graphic interface, following the provider’s MEG or EEG sensor alignment guidelines. Once the corrections for “artifactual” lead field cases were completed, we fed back the flagged artifactual cases to the lead field calculation module for recalculation.

The third and last correction utilized a critical distance value when a lead field lopped within the two previous quality control stages was still flagged as “artifactual. This critical value is a default CiftiStorm parameter applied to the surface-surface distances within the head model, considering the source model distance to the inner skull surface. The critical value can be modified iteratively within the quality control loop until the lead field is checked as “acceptable. Such a critical distance artifact mainly affects EEG head models and lead field, as stated before, due to the type of numerical integration methods for the Poisson equation of electromagnetism in heterogeneous media (Hamalainen and Sarvas, 1989; Hämäläinen et al., 1993; Riera and Fuentes, 1998). This type of artifact is not very common for MEG lead fields obtained by other methods (Huang et al., 1999). The kind of quality control loop and corrections features introduced mainly respond to the GBC EEG challenge and are unique CiftiStorm features.

CiftiStorm can be potentially applied to investigate spectral differences between the MEG and EEG sources further, as in our previous study (Riaz et al., 2021). This study was conducted end-to-end by CiftiStorm to assess the differences in inverse solutions methods, including SSSBL, HIGGS, eLORETA, and LCMV. From this assessment, the corrections were applied to our inverse solutions for non-linear deformations across the source spectra. However, linear methods such as eLORETA or LCMV that do not produce this artifact lead to dissimilar spatial distributions of the MEG and EEG source spectra.

Another CiftiStorm application was the study of spectral source connectivity measures derived from the SSSBL method applied to individuals at risk of cognitive decline (González-López et al., 2022). This study reported meaningful and statistically significant source connectivity changes even when SSSBL was used to obtain inverse solutions from a 10–20 EEG system (19 sensors) commonly found in clinical settings.

The first CiftiStorm release included the forward model pipeline and quality control loop employed for our former lead field study. This release incorporated several new functionalities, such as the graphic interface of the quality control loop and batch processes of the structural and forward model pipelines. We have also increased the number of settings that can be customized for specific databases in “.json” configuration files. Another increment is exploration tools and the quality control loop extension for structural outputs at any stage. This quality control can be applied to the outputs of other third-party CiftiStorm functions such as HCP, Ciftify, FreeSurfer, and FSL, and customization can be used to improve subsequent reconstruction of the source and head models. Additional functionalities, such as quality control of the source model and FSAverage registration, were included to optimize this pipeline. Different MATLAB standard processing tools, such as FieldTrip and SPM, were extremely useful in developing these functionalities. Users can also perform geometric optimization of the source and head models according to each database’s artifacts and follow different processing paths in the pipeline. Direct interaction through an open parametrization of the functions for lead field computations can be obtained with SPM, Brainstorm, FieldTrip, and OpenMEEG or DUNEuro.

CiftiStorm has produced good-quality results in several legacy datasets, including sMRI, MEG, and EEG. However, this pipeline still requires testing with several configurations not considered in this work and might be relevant for real studies. We must test our pipeline in different MEG datasets to perfect the automatic correction of the individual MEG helmet registration. Another limitation is the automatic correction when the image segmentation produces overlapping between surfaces, producing structural errors and low correlation in the sources. We also prepared a release with HCP-compatible and analogous electrophysiological source imaging and connectivity data obtained with our pipeline for the HCP MEG and the CHBP EEG. Future work should include a statistical module in our ESI pipeline to allow users to get group analysis between subjects and datasets.

## 5 Conclusion

This study addresses the pressing challenges for broad EEG electrophysiological source imaging (ESI) integration within the Human Connectome Project (HCP) research framework. We introduced the “CiftiStorm” ESI pipelines, an HCP megconnectome compliant in the Brainstorm suite, which responds directly to the call set by the Global Brain Consortium (GBC) for enhanced EEG ESI reproducibility toward connectomics. By meticulously examining the critical ESI quality factors, including structural, forward model, and inverse model processing, CiftiStorm introduced a high-throughput ESI device. Our pipeline demonstrated remarkable success in achieving high-quality forward model outputs through rigorous quality control and geometrical corrections that targeted the source, head model, and lead field separately. Incorporating a sophisticated cross-spectral inverse solution within an inverse modeling methodology derived from variable resolution electromagnetic tomographic analysis (VARETA) and rigorous mathematical and geometrical prior models, we obtain, for the first time, highly similar MEG/EEG sources, paving the way for more robust and reproducible EEG ESI outcomes in the realm of multimodal neuroimaging.

## Data availability statement

The original contributions presented in the study are included in the article/Supplementary material, further inquiries can be directed to the corresponding author.

## Author contributions

AA-G and DP-L contributed equally to the design, implementation, and manuscript for the structural, forward, and inverse model pipelines and their modules. UR and FR contributed to the forward model pipeline’s lead field numerical accuracy index and quality control loop module. YW and ML contributed to the MEG, EEG preprocessing module. JB-B and EG-M contributed to the structural, forward, and inverse model pipelines. LBCC contributed to the structural pipeline and quality control. GBC contributed to the guidelines, design of all pipelines, and data collection. CHBMP contributed to data collection. MO-O contributed to the structural pipeline. LG-G contributed to the statistical analysis of the pipelines. EM-M contributed to the forward model and inverse model pipelines. LM, MV-S, and MB-V contributed to the preparation of this manuscript. PV-S contributed to guiding the preparation of this study end-to-end and establishing guidelines as GBC co-chairperson. All authors contributed to the article and approved the submitted version.

## Group members of Lifespan Brain Chart Consortium (LBCC), Global Brain Consortium (GBC), and Cuban Human Brain Mapping Project (CHBMP)

Sophie Adler, Aaron Alexander-Bloch, Evdokia Anagnostou, Kevin Anderson, Annemieke Apergis-Schoute, Ariosky Areces-Gonzalez, Duncan Astle, Bonnie Auyeung, Muhammad Ayub, Jong Bin Bae, Gareth Ball, Paula Banca, Simon Baron-Cohen, Richard Beare, Saashi Bedford, Vivek Benegal, Jonathan Berken, Richard Bethlehem, Frauke Beyer, Marjan Biria, John Blangero, Manuel Blesa Cábez, James Boardman, Matthew Borzage, Jorge Bosch-Bayard, Niall Bourke, Matthew Buczek, Edward Bullmore, Vince Calhoun, Mallar Chakravarty, Christina Chen, Casey Chertavian, Gaël Chetelat, Yap Chong, Aiden Corvin,Manuela Costantino, Eric Courchesne, Fabrice Crivello, Vanessa Cropley, Jennifer Crosbie, Nicolas Crossley, Kat Cyr, Anthony David, Marion Delarue, Richard Delorme, Sylvane Desrivieres, Gabriel Devenyi, Maria Di Biase, Ray Dolan, Kirsten Donald, Gary Donohoe, Lena Dorfschmidt, Katharine Dunlop, Anthony Edwards, Jed Elison, Cameron Ellis, Jeremy Elman, Lisa Eyler, Damien Fair, Eric Feczko, Paul Fletcher, Peter Fonagy, Carol Franz, Lidice Galan-Garcia, Margaret Gardner, Ali Gholipour, Jay Giedd, John Gilmore, Juan Domingo Gispert López, David Glahn, Ian Goodyer, P. Grant, Nynke Groenewold, Faith Gunning, Raquel Gur, Ruben Gur, Christopher Hammill, Oskar Hansson, Trey Hedden, Andreas Heinz, Richard Henson, Katja Heuer, Jacqueline Hoare, Bharath Holla, Avram Holmes, Hao Huang, Kiho Im, Jonathan Ipser, Clifford Jack Jr, Andrea Jackowski, Tianye Jia, David Jones, Peter Jones, Benjamin Jung, Eren Kafadar, Rene Kahn, Shivaram Karandikar, Hasse Karlsson, Linnea Karlsson, Ryuta Kawashima, Elizabeth Kelley, Silke Kern, Ki-Woong Kim, Manfred Kitzbichler, William Krsemen, François Lalonde, Brigitte Landeau, Jason Lerch, John Lewis, Jiao Li, Wei Liao, Conor Liston, Michael Lombardo, Pedro Luque Laguna, Jinglei Lv, Briana Macedo, Travis Mallard, Ayan Mandal, Machteld Marcelis, Samuel Mathias, Bernard Mazoyer, Philip McGuire, Michael Meaney, Andrea Mechelli, Laura Mercedes, Harri Merisaari, Kate Merritt, Bratislav Misic, Marcella Montagnese, Sarah Morgan, David Mothersill, Cynthia Ortinau, Rik Ossenkoppele, Minhui Ouyang, Eva Palacios Martínez, Lena Palaniyappan, Leo Paly, Pedro Pan, Christos Pantelis, Min Tae Park, Tomas Paus, Zdenka Pausova, Deirel Paz-Linares, Alexa Pichet Binette, Karen Pierce, Smrithi Prem, Elmo Pulli, Xing Qian, Anqi Qiu, Armin Raznahan, Timothy Rittman, Trevor Robbins, Amanda Rodrigue, Caitlin Rollins, Rafael Romero-Garcia, Lisa Ronan, Monica Rosenberg, David Rowitch, Giovanni Salum, Theodore Satterthwaite, H. Lina Schaare, Russell Schachar, Michael Schöll, Aaron Schultz, Jakob Seidlitz, Zhiqiang Sha, David Sharp, Russell Shinohara, Ingmar Skoog, Christopher Smyser, Reisa Sperling, Dan Stein, Aleks Stolicyn, John Suckling, Gemma Sullivan, Kevin Sun, Benjamin Thyreau, Roberto Toro, Nicolas Traut, Kamen Tsvetanov, Elise Turk, Nicholas Turk-Browne, Jetro Tuulari, Christophe Tzourio, Étienne Vachon-Presseau, Mitchell Valdes-Sosa, Pedro Valdes-Sosa, Sofie Valk, Therese van Amelsvoort, Simon Vandekar, Lana Vasung, Petra Vértes, Lindsay Victoria, Sylvia Villeneuve, Arno Villringer, Jacob Vogel, Konrad Wagstyl, Yin-Shan Wang, Simon Warfield, Varun Warrier, Eric Westman, Margaret Westwater, Heather Whalley, Simon White, Remo Williams, A. Veronica Witte, Ning Yang, B.T. Thomas Yeo, Hyuk Jin Yun, Andrew Zalesky, Heather Zar, Anna Zettergren, Juan Zhou, Hisham Ziauddeen, Dabriel Zimmerman, Andre Zugman, Xi-Nian Zuo.
