# Correction: CiftiStorm pipeline: facilitating reproducible EEG/MEG source connectomics

**DOI:** 10.3389/fnins.2025.1769423

**Published:** 2026-01-19

**Authors:** Ariosky Areces-Gonzalez, Deirel Paz-Linares, Usama Riaz, Ying Wang, Min Li, Fuleah A. Razzaq, Jorge F. Bosch-Bayard, Eduardo Gonzalez-Moreira, Lifespan Brain Chart Consortium (LBCC), Global Brain Consortium (GBC), Cuban Human Brain Mapping Project (CHBMP), Marlis Ontivero-Ortega, Lidice Galan-Garcia, Eduardo Martínez-Montes, Ludovico Minati, Mitchell J. Valdes-Sosa, Maria L. Bringas-Vega, Pedro A. Valdes-Sosa

**Affiliations:** 1 The Clinical Hospital of Chengdu Brain Sciences Institute, School of Life Science and Technology, University of Electronic Science and Technology of China, Chengdu, China; 2 School of Technical Sciences, University “Hermanos Saiz Montes de Oca” of Pinar del Río, Pinar del Rio, Cuba; 3 Department of Neuroinformatics, Cuban Neurosciences Center, Havana, Cuba; 4 Hangzhou Dianzi University, Hangzhou, Zhejiang, China; 5 McGill Centre for Integrative Neurosciences MCIN, LudmerCentre for Mental Health, Montreal Neurological Institute, McGill University, Montreal, QC, Canada; 6 Center for Biomedical Imaging and Neuromodulation, Nathan Kline Institute for Psychiatric Research, Orangeburg, NY, United States; 7 Center for Mind/Brain Sciences (CIMeC), University of Trento, Trento, Italy

**Keywords:** human connectome project, megconnectome, Brainstorm, Ciftify, VARETA, SSSBL, HIGGS, forward model

Lifespan Brain Chart Consortium (LBCC) was included in the author list of the published article; however, the individual consortium member names were omitted from the expandable consortium author list.

The correct consortium author list is provided below:

Sophie Adler, Aaron Alexander-Bloch, Evdokia Anagnostou, Kevin Anderson, Annemieke Apergis-Schoute, Ariosky Areces-Gonzalez, Duncan Astle, Bonnie Auyeung, Muhammad Ayub, Jong Bin Bae, Gareth Ball, Paula Banca, Simon Baron-Cohen, Richard Beare, Saashi Bedford, Vivek Benegal, Jonathan Berken, Richard Bethlehem, Frauke Beyer, Marjan Biria, John Blangero, Manuel Blesa Cábez, James Boardman, Matthew Borzage, Jorge Bosch-Bayard, Niall Bourke, Matthew Buczek, Edward Bullmore, Vince Calhoun, Mallar Chakravarty, Christina Chen, Casey Chertavian, Gaël Chetelat, Yap Chong, Aiden Corvin, Manuela Costantino, Eric Courchesne, Fabrice Crivello, Vanessa Cropley, Jennifer Crosbie, Nicolas Crossley, Kat Cyr, Anthony David, Marion Delarue, Richard Delorme, Sylvane Desrivieres, Gabriel Devenyi, Maria Di Biase, Ray Dolan, Kirsten Donald, Gary Donohoe, Lena Dorfschmidt, Katharine Dunlop, Anthony Edwards, Jed Elison, Cameron Ellis, Jeremy Elman, Lisa Eyler, Damien Fair, Eric Feczko, Paul Fletcher, Peter Fonagy, Carol Franz, Lidice Galan-Garcia, Margaret Gardner, Ali Gholipour, Jay Giedd, John Gilmore, Juan Domingo Gispert López, David Glahn, Ian Goodyer, P. Grant, Nynke Groenewold, Faith Gunning, Raquel Gur, Ruben Gur, Christopher Hammill, Oskar Hansson, Trey Hedden, Andreas Heinz, Richard Henson, Katja Heuer, Jacqueline Hoare, Bharath Holla, Avram Holmes, Hao Huang, Kiho Im, Jonathan Ipser, Clifford Jack Jr, Andrea Jackowski, Tianye Jia, David Jones, Peter Jones, Benjamin Jung, Eren Kafadar, Rene Kahn, Shivaram Karandikar, Hasse Karlsson, Linnea Karlsson, Ryuta Kawashima, Elizabeth Kelley, Silke Kern, Ki-Woong Kim, Manfred Kitzbichler, William Krsemen, François Lalonde, Brigitte Landeau, Jason Lerch, John Lewis, Jiao Li, Wei Liao, Conor Liston, Michael Lombardo, Pedro Luque Laguna, Jinglei Lv, Briana Macedo, Travis Mallard, Ayan Mandal, Machteld Marcelis, Samuel Mathias, Bernard Mazoyer, Philip McGuire, Michael Meaney, Andrea Mechelli, Laura Mercedes, Harri Merisaari, Kate Merritt, Bratislav Misic, Marcella Montagnese, Sarah Morgan, David Mothersill, Cynthia Ortinau, Rik Ossenkoppele, Minhui Ouyang, Eva Palacios Martínez, Lena Palaniyappan, Leo Paly, Pedro Pan, Christos Pantelis, Min Tae Park, Tomas Paus, Zdenka Pausova, Deirel Paz-Linares, Alexa Pichet Binette, Karen Pierce, Smrithi Prem, Elmo Pulli, Xing Qian, Anqi Qiu, Armin Raznahan, Timothy Rittman, Trevor Robbins, Amanda Rodrigue, Caitlin Rollins, Rafael Romero-Garcia, Lisa Ronan, Monica Rosenberg, David Rowitch, Giovanni Salum, Theodore Satterthwaite, H. Lina Schaare, Russell Schachar, Michael Schöll, Aaron Schultz, Jakob Seidlitz, Zhiqiang Sha, David Sharp, Russell Shinohara, Ingmar Skoog, Christopher Smyser, Reisa Sperling, Dan Stein, Aleks Stolicyn, John Suckling, Gemma Sullivan, Kevin Sun, Benjamin Thyreau, Roberto Toro, Nicolas Traut, Kamen Tsvetanov, Elise Turk, Nicholas Turk-Browne, Jetro Tuulari, Christophe Tzourio, Étienne Vachon-Presseau, Mitchell Valdes-Sosa, Pedro Valdes-Sosa, Sofie Valk, Therese van Amelsvoort, Simon Vandekar, Lana Vasung, Petra Vértes, Lindsay Victoria, Sylvia Villeneuve, Arno Villringer, Jacob Vogel, Konrad Wagstyl, Yin-Shan Wang, Simon Warfield, Varun Warrier, Eric Westman, Margaret Westwater, Heather Whalley, Simon White, Remo Williams, A. Veronica Witte, Ning Yang, B.T. Thomas Yeo, Hyuk Jin Yun, Andrew Zalesky, Heather Zar, Anna Zettergren, Juan Zhou, Hisham Ziauddeen, Dabriel Zimmerman, Andre Zugman, Xi-Nian Zuo.

The original version of this article has been updated.

