# Supplementary material for: CiftiStorm pipeline: facilitating reproducible EEG/MEG source connectomics
Source: Front Neurosci. 2024 Apr 12;18:1237245. doi: 10.3389/fnins.2024.1237245 (PMC11047451; doi:10.3389/fnins.2024.1237245)
Supplement: Supplementary file 1 [file Table_1.DOCX]

Supplementary Material

# Exploratory processing in multinational Datasets

We have performed the initial test of concept for the structural processing and head modeling in three public datasets: Human Connectome Project (HCP) ^[23]^, Cuban Human Brain Mapping (CHBMP) ^[24]^ and Healthy Brain Network (HBN) ^[25]^. The raw and processed data for these databases, amongst others, is being curated in the CCLab high performance computer. The same data is mirrored in Compute Canada and will also be mirrored in Cuba, in the near future, as part of the Collaborative effort carried out by CCLab in the Cuba-China-Canada project. These are part of the set of databases stored in the CCLab high performance computer, which contain several modalities that we summarize in Table 1, where we have highlighted in gray color the databases used for the initial test of concept.

Table 1 Curated databases of which physical copies are mirrored in UESTC, Cuban Neuroscience Center, and Compute Canada

| **DATASET** | **N** | **EEG** | **MEG** | **MRI** | **fMRI** | **DTI** | **Behavior/ clinical** | **Size** |
| --- | --- | --- | --- | --- | --- | --- | --- | --- |
| HCP | 900 |  | 95 | 813 | 889 | 889 |  | 79TB |
| CMI | 1317 | 1310 |  | 1380 |  | 516 |  | 11TB |
| CHBM | 282 | 177 |  | 282 |  | 177 | 88 | 29TB |
| CAMCAN | 700 |  | 647 | 653 | 650 | 642 |  | 2TB |
| Barbados | 520 | 108&95 |  |  |  |  | Soft Neurological Signs, IQ WASI CAARS Eco quest. | 1.6GB |
| China | 100 |  |  |  |  |  | Audio stimulus | 182GB |
| Omega | 184 | 184 |  | 184 |  |  | Questionnaire | 367GB |
| Parkinson-CIREN | 26 | 26 |  |  |  |  | UPDRS, Cognitive batteries | 943MB |
| Colombia | 45 | 45 |  |  |  |  | MMSE | 76GB |
| PPMI | 2276 |  |  | 1157 | 195 | 855 |  | 356GB |

We illustrate the processing workflow for the HBN dataset, which was also replicated for the HCP and CHBM datasets. See in Figure 1 the steps followed in processing the HBN database in collaboration with the Child Mind Institute (CMI), where only the T1 MRI was acquired consistently with the EEG recordings. For this reason, the test of the concept followed, as a general rule, the HCP-like pipeline branch designed for legacy data (Freesurfer and Ciftify).


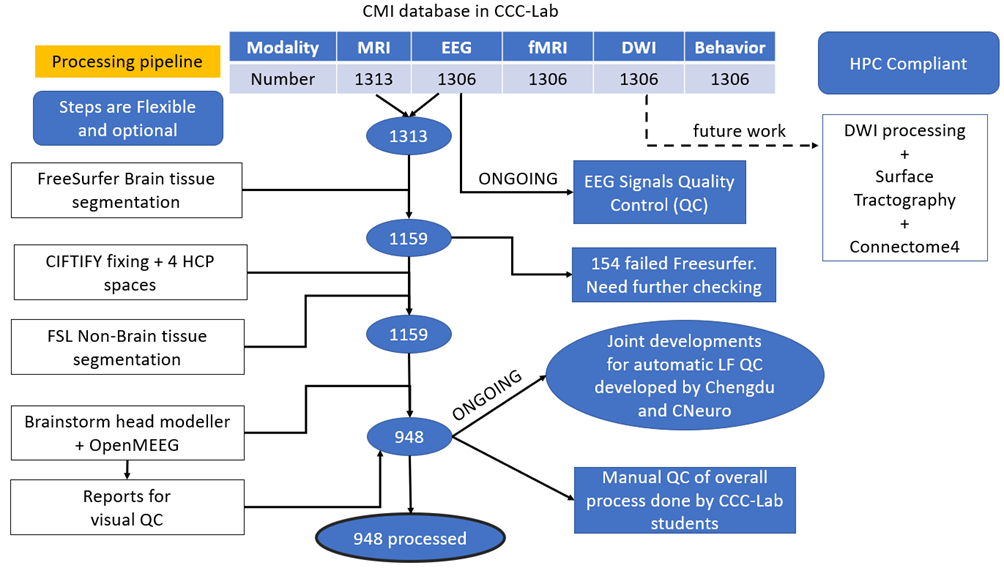
The purpose of the HBN dataset was to investigate brain connectivity and its longitudinal association with clinical and behavioral data. Attempting to reproduce similar HCP standards. Several neuroimaging modalities (MRI, EEG, fMRI, DWI) and behavioral/clinical data were collected in this database and for large number of subjects. However, by manual inspection of some of their structural MRI acquisitions and the HCP structural and head modeling outputs produced by our pipelines, we have found that the HBN database quality falls far behind the HCP database standards.

Figure 1 Illustration of the processing steps followed by our pipeline in the Healthy Brain Networks dataset.

We now emphasize that our objective is to produce the space features based on these multiple modalities and to facilitate cross-modality comparison, following the high-quality HCP-like processing and through the registration FSAverage space. Notably, the HCP database, although its current release includes the MRI structural outputs that were obtained using the HCP pipelines for MRI T1 and T2, we have produced a sub-data using the processing pipeline for MRI T1 alone based on Freesurfer and Ciftify. This was done to evaluate the differences between the MRI T1 (legacy) and MRI T1/T2 processing paths and to obtain outputs that are analogous to the legacy HBN and CHBMP databases. It was confirmed through manual inspection that the structural results produced by the T1 alone pipeline (Freesurfer and Ciftify) render similar standards to the structural outputs produced by the HCP T1 and T2 pipeline, given in terms of the brain tissue segmentation.

We have found large artifacts caused by movement and noise in both the T1 and T2 MRIs of the HBN database. For most subjects, the artifacts were quite extended spatially, covering both external and tissue areas. This makes it challenging to judge the cost/benefit relationship in the decision to remove it using any preprocessing. As we explain in the next section, any preprocessing would lead to hollowed MRIs or imperfect segmentation.

# CiftiStorm visual interface and configuration

We have developed a suit for the friendly interaction with the HCP structural pipeline, which is executed by calling the function (ciftistorm), see Figure 2. This is an open-source suit, written for MATLAB R2020, which can be run in Windows and Linux systems and evokes private dependencies of the HCP pipelines of Brainstorm. If not evoked directly from the pipeline, outputs from the structural HCP pipeline are required before any processing, such as HCP-compatible structural elements (MRI gray matter and head segmentations and atlases) obtained from the MRI preprocessing in two alternative modes.

Mode 1) T1w alone from CIFTIFY preprocessing.

Mode 2) T1w and T2w from preprocessing with the HCP pipeline.

Mode 3) Anatomical template based on Brainstorm.


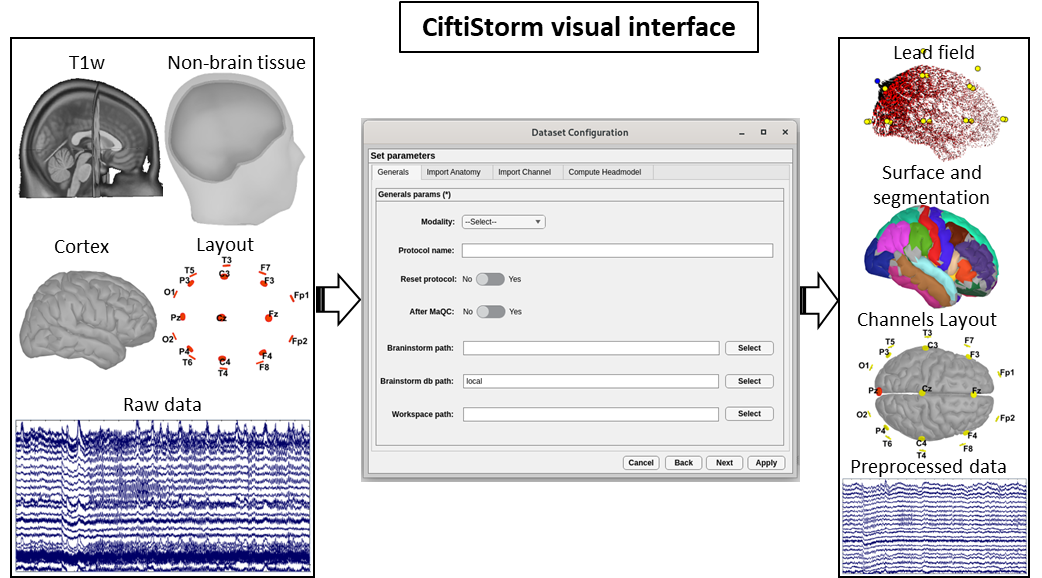
In addition, information relative to the recordings is necessary (in Brainstorm) to incorporate the sensor layout that is used for the construction of the head model and calculation of the lead field. Also, the (MEE or EEG) signals if specific preprocessing of the data is desired.

Figure 2 Visual interface of input and output data representation for HCP and BST source head modeler (CiftiStorm) toolbox.

The processing, which can be configured by the user in the interface shown in Figure 2 center, provides visuals about the head model, the cortical segmentation and atlas, the co-registration of the sensors in the scalp surface, the preprocessed data (MEG/EEG) if needed and the MEG/EEG lead fields. These outputs, shown in Figure 2 right, are stored in a standard Brainstorm format that can be read later to analyze electrophysiological source imaging and connectivity by the BC-VARETA. The visual interface of the pipeline comprises four tabs with configurable elements that are required for the processing.


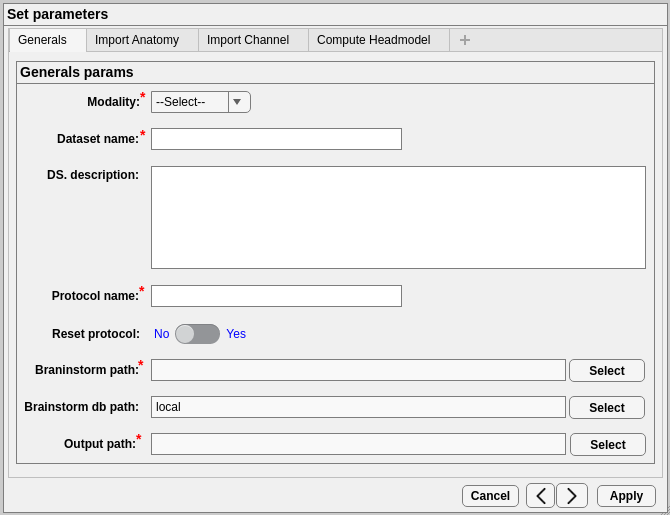
Tab 1) Contains 1) General parameters (Figure 3 and Table 2) that require parameters such as Modality, Dataset name, Dataset description, Protocol name, Brainstorm local path, Brainstorm database path, and the paths to store the outputs Output path. See in Table 2 the description for the fields in the general parameters tab included in Figure 3.

Figure *3* General params tab in the CiftiStorm toolbox

Table 2 Description of general parameters in HCP BST source head modeler

| **General Tab** | | | | |
| --- | --- | --- | --- | --- |
| **Index** | **Name** | **Values** | **Default value** | **Description** |
| 1 | General params | | | |
| 1.1 | Modality | EEG, MEG | --Select-- | Data modality for the process |
| 1.2 | Dataset name | test | (empty) | Name of the datasets to be processed |
| 1.3 | Dataset description | Text | (empty) | Description of the dataset to be processed |
| 1.4 | Protocol name | text | (empty) | Name to use as a template in the Brainstorm protocols |
| 1.5 | Reset protocol | No/Yes | Yes | Reset the Brainstorm protocol if it exist in the Brainstorm Database |
| 1.6 | Brainstorm path | Folder location | (empty) | Root path folder of Brainstorm toolbox |
| 1.7 | Brainstorm db path | Folder location | “local” | Root path folder for the Brainstorm database. The default value is “local”. This option will put the database address in the user directory |
| 1.8 | Output path | Folder location | (empty) | Root path folder to store the processing results |
| 4 | Cancel | | | |
| 5 | Apply | | | |

Tab 2) Contains 1) The anatomical structure used to define the head and source model (Figure 4 and Table 3), which includes 1.1) Default anatomy provided by Brainstorm to be used to compute the head model, source model, and lead field in case the subject T1 is not available, that include the anatomy template name and the default atlas to be used. 1.2) The path to the HCP individual anatomy includes the anatomy path, the T1w image name, and the atlas to be used. 2) Common parameters to be used in the import anatomy process such as 2.1) Layer descriptor (so far, we include one surface processing in our pipeline), 2.2) the number of vertices in the geometry of the head mesh, skull mesh, and source model (cortex), 2.3) the path of the non-brain surface to register with the subject sMRI and 2.4-5) the MRI transformation path and file in case of need in the processing. In Table 3, we include the description for the fields in the structural parameters tab in Figure 4.


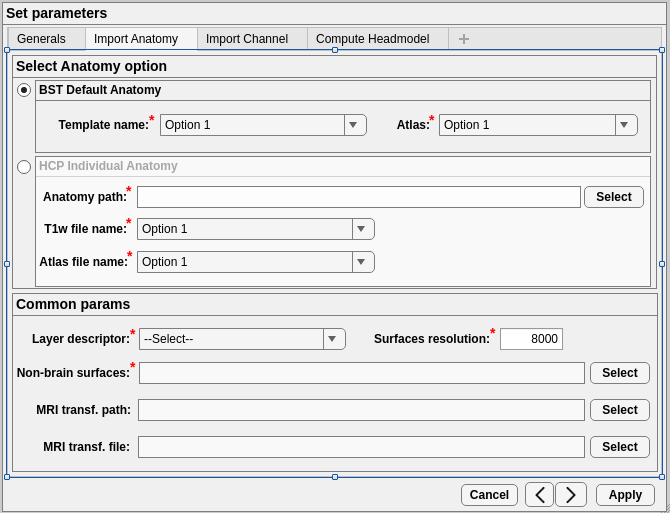
Table 3 HCP BST source head modeler anatomical params description

Figure *4* Anatomical params tab in the CiftiStorm toolbox.

| **Anatomical structure Tab** | | | | |
| --- | --- | --- | --- | --- |
| **Index** | **Name** | **Values** | **Default** | **Description** |
| 1 | Anatomy configuration | | | |
| 1.1 | BST default (Template name) | ICBM152, Colin27, Nonalign | --Select-- | This option will use a default Brainstorm anatomical template. |
| 1.2 | BST template atlas |  | --Select-- | Atlas included in the selected BST anatomy template |
| 1.2 | HCP individual Anatomy | | | |
| 1.2.1 | Anatomy path | Folder location | (empty) | Root folder that contains all subjects with HCP format anatomy |
| 1.2.2 | T1w file name | text | --Select-- | The T1w file name is included in the structural anatomy folder |
| 1.2.3 | Atlas file name | text | --Select-- | The Atlas file name is included in the structural anatomy folder |
| 2 | Surface resolution | | | |
| 2.1 | Layer descriptor | Pial, Midthickness, white | midthickness | The brain’s surface to be used in the processing |
| 2.2 | Surfaces resolution | 5000-15000 | 8000 | Resolution for the head, skull, and cortex surfaces to downsample after importing it in the structural pipeline |
| 2.3 | Non-brain surfaces path | Folder location | (empty) | The Root folder containing the non-brain tissues for each subject included in the “Anatomy path.” |
| 2.4 | MRI transformation path | Folder location | (empty) | The folder location for the MRI transformation in case it is needed. |
| 2.5 | MRI transformation file | File location | (empty) | The reference path location for the MRI transformation file depends on 2.4. |
| 3 | Cancel | | | |
| 4 | Apply | | | |


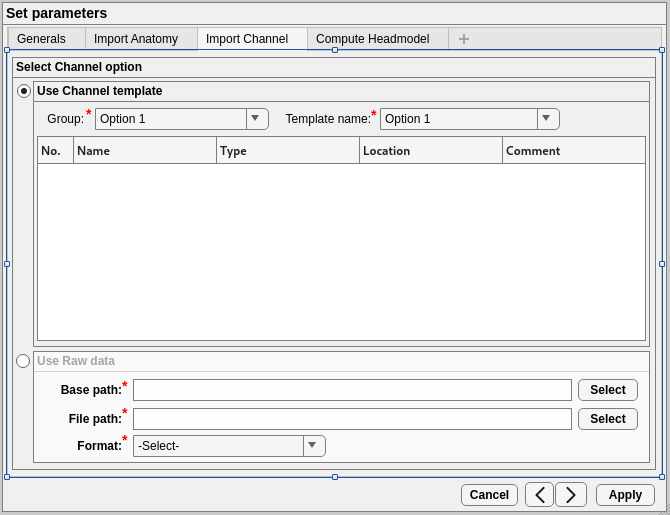
Tab 3) Contains 1) Sensor layout information (Figure 5 and Table 4). Definition of the type of 1.1) template sensor layout for the data and 1.2) specification of labels in the specific system, which can be projected to standard anatomy alternatively. Selection of channels for constructing the head model and computation of lead field. Alternatively, the tab contains 2) the specific sensor layout, which may be included 2.1) in the base path of the raw data, 2.2) the specified path location of the file, and 2.3) the specific file format.

Figure *5* Channel parameters tab in the HCP_BST source head modeler toolbox

Table 4 HCP BST source head modeler anatomical params description

| **Channel Tab** | | | | |
| --- | --- | --- | --- | --- |
| **Index** | **Name** | **Values** | **Default** | **Description** |
| 1 | Use channel template | | | |
| 1.1 | Group | ICBM152, Colin27, Nonalign | --Select-- | Group name of the Brainstorm sensors layout |
| 1.2 | Template name | 10-05, 10-20, etc. | --Select-- | Template name of the Brainstorm sensors layout |
| 2 | Use raw data (Use the raw data information as a sensor layout) | | | |
| 2.1 | Base path | Folder location | (empty) | Root folder for the subject raw data |
| 2.2 | File location | File location | (empty) | File or folder as a reference for the raw data |
| 2.3 | Format | ‘mat’, ’mff’, ’edf’ | --Select-- | Raw data format included in the structural toolbox |
| 2.5 | Individual selection | True or False | True | Select or unselect a specific label from the selected sensor layout |
| 3 | Cancel | | | |
| 4 | Apply | | | |

Tab 4) Contains the specific parameters for the head model and Learfield computation process (Figure 6 and Table 5). 1) Use default parameters, 2) Common parameters, 3) This process includes three methods to select in the processing; 3.1) Overlapping Spheres, 3.2) OpenMEEG BEM or 3.3) DUNEuro FEM .


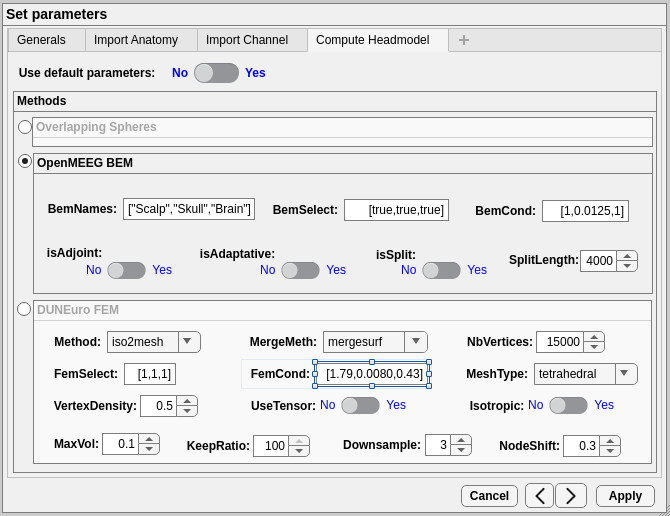


Figure *6* Compute head model tab in the CiftiStorm toolbox

Table 5 Compute head model parameters tab in the CiftiStorm.

| **Compute Head model Tab** | | | | |
| --- | --- | --- | --- | --- |
| **Index** | **Name** | **Values** | **Default** | **Description** |
| 1 | Use raw data | No or Yes | Yes | Use default parameters and method (OpenMEEG BEM) |
| 2 | Methods | | | |
| 2.1 | Overlapping spheres | True/false | false | MEG overlapping sphere forward model |
| 2.2 | OpenMEEG BEM | True/false | true | Call OpenMEEG to compute a BEM solution for Brainstorm |
| 2.2.1 | BEM names | [1, nLayers] | ["Scalp","Skull","Brain"] | Cell array of filenames to use to compute a BEM solution. |
| 2.2.2 | BEM select | Logical [1, nLayers] | [true, true, true] | Logical cell array of filenames to use to compute a BEM solution. |
| 2.2.4 | BemCond | [1, nLayers] | [1,0.0125,1] | Array of layer conductivities |
| 2.2.6 | isAdjoint | True/false | false | If true, use adjoint formulation (less memory, longer) |
| 2.2.7 | isAdptative | True/false | true | If true, use adaptive integration (more accurate, 3x longer) |
| 2.2.8 | isSplit | True/false | false | Process the dipoles by blocks |
| 2.2.9 | SplitLength | numeric | 4000 | If isSplit=true. Number of blocks |
| 2.3 | DUNEuro FEM | True/false | false | Call DUNEuro to compute a FEM solution for Brainstorm. |
| 2.3.1 | Method | Iso2mesh-2021, iso2mesh, Brain2mesh, SimNIBS3, SimNIBS4, ROAST, FieldTrip | Iso2mesh | FEM mesh generation method. See (process_fem_mesh). This function is part of the Brainstorm software: https://neuroimage.usc.edu/brainstorm |
| 2.3.2 | MergeMethod | MergeMesh, MergeSurf | mergesurf | Method to marge the input surfaces |
| 2.3.3 | NbVertices | numeric | 15000 | Number of vertices of the mesh surfaces. |
| 2.3.4 | FemSelect | Logical [1, nMesh] | [1, 1, 1] | Mesh surfaces to use in the processing. |
| 2.3.5 | FemCond | [1, nMesh] | [1.79,0.008,0.43] | FEM mesh conductivity |
| 2.3.6 | Isotropic | True/false | true |  |
| 2.3.7 | MaxVol | double | 0.1 | Max tetrahedral volume (in cm^3) |
| 3 | Cancel | | | |
| 4 | Apply | | | |

# BC-VARETA source spectral processing and structural priors


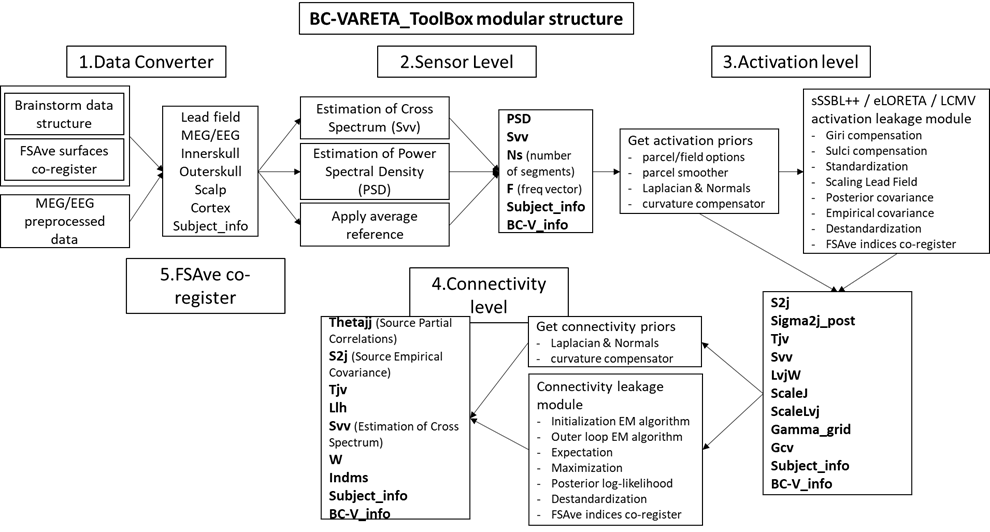
BC-VARETA is programmed in five modules that we illustrate in Figure 7 in a hierarchical order. In Figure 8, we describe the outputs of BC-VARETA, given by modules 2 (sensor level analysis) using the Hilbert envelope of the filtered data and Slepian windows, 3 (activation level analysis) using the spectral Structured Sparse Bayesian Learning (sSSBL) method ^[45]^ and 4 (connectivity level analysis) the Hidden Gaussian Graphical State-Model (HIGGS) with connectivity regularization of the Hermitian Graphical LASSO (hgLASSO) method ^[46]^.

Figure *7* Modular structure of BC-VARETA Toolbox.

Module 1) Converter for files from HCP structural and head modelling pipeline and MEG/EEG data to be loaded in BC-VARETA analysis.

Module 2) Sensor level analysis via the FFT of the MEG/EEG signals to produce the cross-spectra, as well as the spectra and specific band topographies for quality control.

Module 3) Activation level analysis via sSSBL spectral electrophysiological source imaging to obtain the MEG/EEG source spectra.

Module 4) Connectivity level analysis via HIGGS spectral electrophysiological source connectivity.

Module 5) Registration to common structural space via the interpolation of the low-resolution cortex discretization for the individual subject.

This process can be carried out by the combination of different methods to obtain the activations in module 2, through eLORETA and LCMV referred above, and to obtain the connectivity in module 3, through the Hermitian Graphical Ridge (hgRidge) and the Hermitian Graphical Naïve (hg-Naïve) estimators.


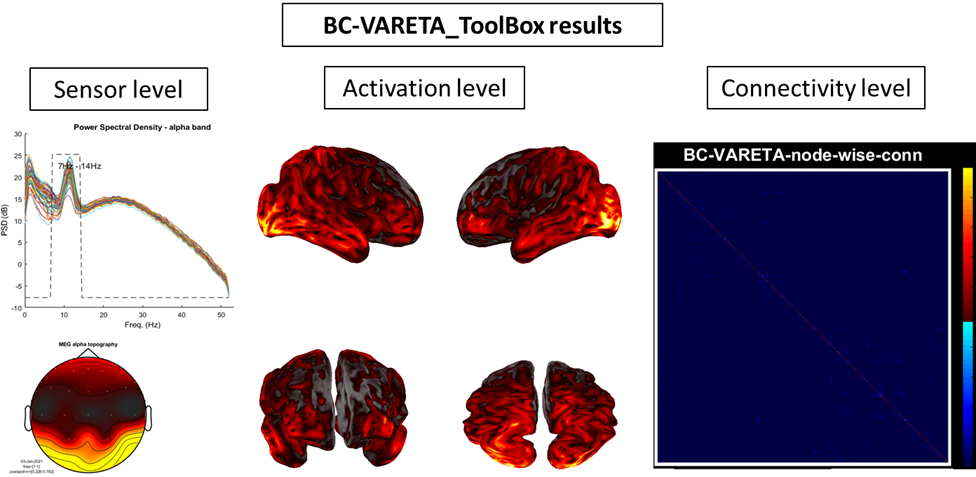
Using the methods sSSBL for 3 (activation level) and HIGGS/hgLASSO for 4 (connectivity level), which we illustrated in Figure 8, is the optimal setup for the BC-VARETA toolbox since these provide statistical guarantees, which we explain here briefly.

Figure *8* BC-VARETA outputs for the alpha band with one of our methods

An unbiased level of sparsity was obtained for sSSBL with the F-score computed from the rate between the explained variances ${\hat{\mathbf{s}}}_{\boldsymbol{\iota}}^{2}\left( \nu\right)$ and residual variances ${\check{\boldsymbol{\sigma}}}_{\boldsymbol{\iota}}^{2}\left( \nu\right)$ of the posterior distribution $N^{\mathcal{C}}\left( \boldsymbol{\iota}_{\mathcal{m}}\left( \nu\right) | {\hat{\boldsymbol{\iota}}}_{\mathcal{m}}\left( \nu\right),{\check{\boldsymbol{\Sigma}}}_{\boldsymbol{\iota\iota}}\left( \nu\right) \right)$ for the mixed effect model of the true sources ^[23]^:

$\boldsymbol{\iota}_{\mathcal{m}}\left( \nu\right)={\hat{\boldsymbol{\iota}}}_{\mathcal{m}}\left( \nu\right)+\boldsymbol{\zeta}_{\mathcal{m}}\left( \nu\right)$ (3-1)

where the explained variances are computed as ${\hat{\mathbf{s}}}_{\boldsymbol{\iota}}^{2}\left( \nu\right)=diag({\hat{\mathbf{S}}}_{\boldsymbol{\iota\iota}}\left( \nu\right))$ with ${\hat{\mathbf{S}}}_{\boldsymbol{\iota\iota}}\left( \nu\right)=\left( 1/m \right)\sum_{i=1}^{m} {\hat{\boldsymbol{\iota}}}_{\mathcal{m}}\left( \nu\right){\hat{\boldsymbol{\iota}}}_{\mathcal{m}}^{\dagger}$, the residual variances ${\check{\boldsymbol{\sigma}}}_{\boldsymbol{\iota}}^{2}\left( \nu\right)=diag({\check{\boldsymbol{\Sigma}}}_{\boldsymbol{\iota\iota}}\left( \nu\right))$ with ${\check{\boldsymbol{\Sigma}}}_{\boldsymbol{\iota\iota}}\left( \nu\right)$ the estimator of the posterior covariance. The F-score is $F={{\hat{\mathbf{s}}}_{\boldsymbol{\iota}}^{2}\left( \nu\right)}/{{\check{\boldsymbol{\sigma}}}_{\boldsymbol{\iota}}^{2}\left( \nu\right)}>1$, which tests the hypothesis that the fixed effect ${\hat{\boldsymbol{\iota}}}_{\mathcal{m}}\left( \nu\right)$ is larger than the residuals $\boldsymbol{\zeta}_{\mathcal{m}}\left( \nu\right)$.

HIGGS produces unbiased estimation of the connectivity. In the first place, it eliminates the bias of $\boldsymbol{\Theta}_{\boldsymbol{\iota\iota}}\left( \nu\right)$ via “de-sparsification”:

$\left( {\hat{\boldsymbol{\Theta}}}_{\boldsymbol{\iota\iota}}^{\left( k+1 \right)}\left( \nu\right) \right)_{unb}\leftarrow2{\hat{\boldsymbol{\Theta}}}_{\boldsymbol{\iota\iota}}^{\left( k+1 \right)}\left( \nu\right)-{\hat{\boldsymbol{\Theta}}}_{\boldsymbol{\iota\iota}}^{\left( k+1 \right)}\left( \nu\right){\check{\boldsymbol{\Psi}}}_{\boldsymbol{\iota\iota}}^{\left( k \right)}\left( \nu\right){\hat{\boldsymbol{\Theta}}}_{\boldsymbol{\iota\iota}}^{\left( k+1 \right)}\left( \nu\right)$ (3-2)

This is an extrapolation to the complex domain of the unbiased estimator for the Graphical LASSO theory of Jankova and Van De Geer. Debiasing $\boldsymbol{\Theta}_{\boldsymbol{\iota\iota}}\left( \nu\right)$ is particularly relevant to produce reliable neural connectivity estimates that depend on it. This debiased estimator has an additional advantage, it allows us to carry thresholding of the connectivity to obtain reliable sparse estimators.

As it is shown in ^[22]^ the unbiased estimator for HIGGS $\left( {\hat{\boldsymbol{\Theta}}}_{\boldsymbol{\iota\iota}}^{\left( k+1 \right)}\left( \nu\right) \right)$ follows the distribution:

$\left( \hat{\Theta}_{\boldsymbol{\iota\iota}}^{\left( k+1 \right)}\left( \nu;i,j \right) \right)_{unb}\sim N_{1}^{\mathbb{C}}\left( \left( \hat{\Theta}_{\boldsymbol{\iota\iota}}^{\left( k+1 \right)}\left( \nu;i,j \right) \right)_{unb} | \Theta_{\boldsymbol{\iota\iota}}\left( \nu;i,j \right),\frac{\sigma_{ij}}{\sqrt{m}} \right)$ (3-3)

$\sigma_{ij}=\hat{\Theta}_{\boldsymbol{\iota\iota}}^{\left( k+1 \right)}\left( \nu;i,i \right)\hat{\Theta}_{\boldsymbol{\iota\iota}}^{\left( k+1 \right)}\left( \nu;j,j \right)+\hat{\Theta}_{\boldsymbol{\iota\iota}}^{\left( k+1 \right)}\left( \nu;i,j \right)$ (3-4)

with fixed value of the regularization parameter $\alpha_{\boldsymbol{\iota}}=\sqrt{m\log\left( q \right)}$ and $m\gg q$, whose z-statistic which possesses a Rayleigh distribution with variance $1/\sqrt{2}$.

$z\left( \nu;i,j \right)\sim2e^{-z^{2}\left( \nu;i,j \right)}z\left( \nu;i,j \right)$ (3-5)

$z\left( \nu;i,j \right)=\sqrt{\left| {\sqrt{m}\left( \hat{\Theta}_{\boldsymbol{\iota\iota}}^{\left( k+1 \right)}\left( \nu;i,j \right) \right)_{unb}}/{\sigma_{ij}} \right|}$ (3-6)

The values of $\hat{\Theta}_{\boldsymbol{\iota\iota}}^{\left( k+1 \right)}\left( \nu\right)$ with $z\left( \nu;i,j \right)$ lower than a threshold are zeroed to ensure a Family Wise Error of type I. It is to be noted that this debiasing and thresholding yields, in the final iteration, a statistically guaranteed thresholder connectivity matrix.

The analysis in 2 (activation level) and 3 (connectivity level) can also be carried out in multiple combinations of physical priors, which can be evoked for all methods. These are included in json configuration files, along with general configuration and other control parameters, that are depicted in Figure 9. The physical priors refer to parameters organized in two different groups: 1) spectral constraints and 2) gray matter structural constraints.


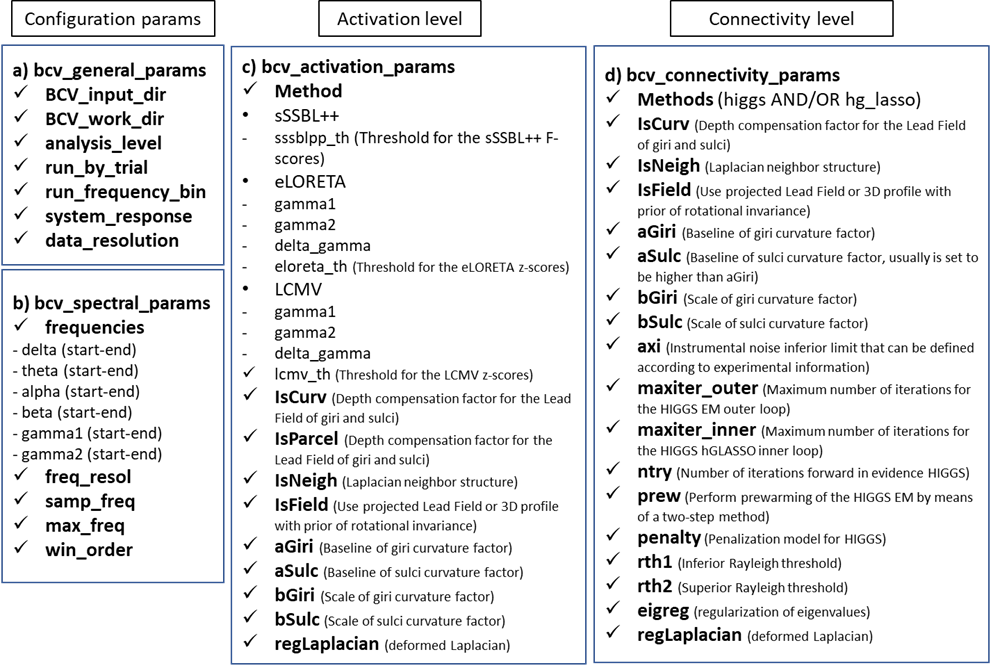
There are four modes of constraints based on the following assumptions for activations or connectivities in relation to different frequencies in the spectra: 1) activations or connectivities are independent for every frequency component. This is the mode run by frequency bin = 1 (for the logical parameter in Figure 9a), which is very consuming in terms of both time and memory 2) activations or connectivities are common for every frequency band. This is the mode run by frequency bin = 0 (for the logical parameter in Figure 9a) using the average cross-spectra of every frequency band (that are defined in Figure 9b), which is more economical in terms of both time and memory, as well as increase the sample number by a factor equal to the number frequency bins in the band 3- the spatial support of the activations and connectivity (nonzero values) is common for all frequencies in the spectra. This is the mode system_response = 1 (for the logical parameter in Figure 9a) that uses the average F-scores for all frequencies, which is devoted to the postprocessing of the connectivity tensor via spectral factorization analysis to obtain the directed connectivity.

Figure *9* BC-VARETA reconfigurable parameters that are included in the json files

There are four modes for the structural constraints based on assumptions for the gray matter spatial distribution of activations and connectivities (Figure 9c and Figure 9d): 1- the scale of activations or connectivities at every point of the cortical surface is linear proportion to the cortical curvature in such a way that their visibility at the gyri is larger than at the sulci. This is the mode IsCurv = 1 (Figure 9c), which performs a linear transformation of the Lead Field with optimal slope and intercept (cross-validated in simulations) that are different for the giri (aGiri, bGiri) and sulci (aSulc, bSulc). 2- there is a high likelihood for the activations or connectivities to reinforce each other in closed regions or parcels, given the functional organization of cortical space. This the mode IsParcel = 1, which uses sparse group penalization of activity and connectivity of the parcels defined within a cortical atlas. 3- there is a high likelihood for neighbor activations or connectivities to reinforce each other, given the large density of lateral connections in the cortex. This the mode IsNeigh = 1, which performs a matrix transformation of the Lead Field by the inverse Laplacian. We use the deformed graph Laplacian with an optimal regularization parameter regLaplacian (cross-validated in simulations). 4- there is a high likelihood for the three components (X, Y, Z) of the activation vector and their connectivity to reinforce each other, given their origin in a common physical magnitude that is described by a vector field. These can be constrained in three different ways that assume different degrees of strength in their coupling: mode IsField = 1, which assumes the fields are generally oriented concerning the cortical surface; IsField = 2, which assumes the field probabilities are rotationally invariant with respect to the surface normal and IsField = 3 that assumes the field probabilities are spherically invariant.

# Validation methods for electrophysiological source imaging and connectivity: concurrency of BC-VARETA in simulations and real data using different head models and lead fields


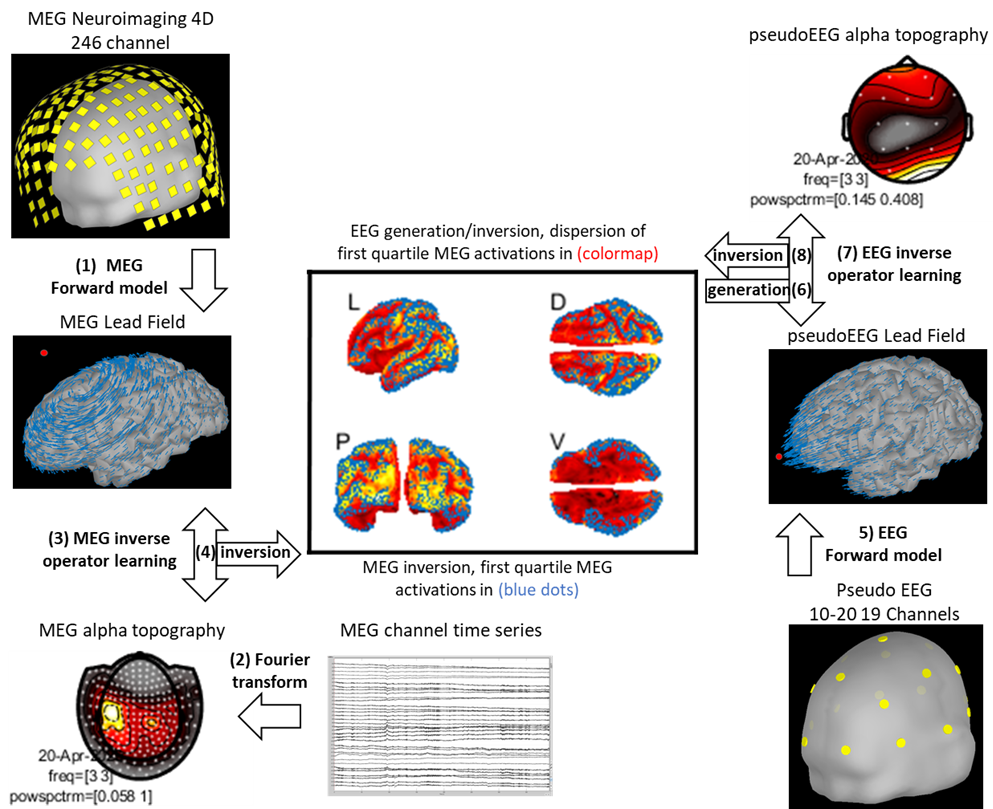
We have designed a simulation package for the validation of Electrophysiological Source Imaging and Connectivity methods. Rather than use an artificial set of simulations, the “ground truth” for the simulations is, in this case, fixed from an ESI solution obtained from a high-density, high-quality real MEG experiment to establish a landmark an expedient that produces lead fields that are better conditioned than those of EEG (Figure 10). This is then used to simulate “pseudo-EEG signals” with a much lower electrode density “and with sources reflected on the scalp with a less well-conditioned EEG forward model defined on the same head geometry. Then, the pseudoEEG is inverted to produce the estimated sources that are compared against the MEG ground truth. The MEG is ground truth obtained by the basic inverse method Minimum Norm ^[68]^, such that, to avoid any type of bias, it does not consider any structural or functional prior.

Figure *10* Illustration pseudoEEG simulation from real MEG sources

Our simulation pipeline follows 9 steps that are shown in Figure 10:

Step 1) Extraction of the MEG head model and lead field following our Brainstorm pipeline for MEG, which was explicitly designed to utilize the HCP structural data, illustrated in Figure 9 for the HCP subject 175237. The head model included one tissue layer (scalp) extracted with FSL and a grid of 8K generators distributed in the middle layer of the cortex.

Step 2) The cross-spectra were obtained at a spectral resolution of 0.5Hz from the Fourier transform (FT) applied to the HCP MEG preprocessed resting data segments recorded at a sampling rate of 508Hz. This yielded, in the calculation of the cross-spectra for each HCP subject, a sample number of 1032 or 172(segments)×6(Slepian windows) per frequency bin.

Step 3) MEG inverse modeling of the source transfer operator via the Minimum Norm method, using the average MEG cross-spectra (from step 2) for each frequency band and Lead Field (from step 1). This process used an optimal selection of the Minimum Norms regularization parameters by cross-validation of the solution that best fits the cross-spectra.

Step 4) Inversion to obtain the MEG source cross-spectra for each band from the left and right cross-product of the Minimum Norms transfer operator by the MEG sensor average cross-spectra.

Step 5) Extraction of the EEG head model corresponded to 19 channels from the 10-20 system, also computed following our Brainstorm pipeline for EEG via the Boundary Elements Method. The head model included three tissue layers (inner skull, outer skull, scalp) extracted with FSL, the same grid of 8K generators distributed in the middle layer of the cortex.

Step 6) Generation of the EEG sensor average cross-spectra for each band from the left and right cross-product of the EEG lead field by the source cross-spectra obtained from MEG.

Step 7) EEG inverse modeling of the source transfer operator via the inverse methods (eLORETA, LCMV, sSSBL), using the average EEG cross-spectra (from step 2) for each frequency band and Lead Field (from step 5). This process used an optimal selection of the Minimum Norms regularization parameters by cross-validation of the solution that best fit the cross-spectra.

Step 8) Inversion to obtain the EEG source cross-spectra for each band from the left and right cross-product of the Minimum Norms transfer operator by the pseudo-EEG sensor average cross-spectra.

This process illustrated in Figure 10 was replicated for the whole HCP MEG data (about 70 subjects), producing analogous outputs for the real MEG and pseudoEEG: head models, lead fields, priors, sensor cross-spectra, as well as the source transfer operator computed with different methods, which is later used to determine the source cross-spectra spectra, and quality measures of the differences between MEG and EEG sources spectra obtained for five spectral bands. We have included in our simulation pipeline three different methods for an initial test of the concept for the quality measures for the spectra, eLORETA, LCMV, and sSSBL. This is available on GitHub (https://github.com/CCC-members/HCP-MEGvsEEG_Concurrency). However, the results or open-source code of our pipeline can be easily extended to 1- include quality measures to compare MEG/EEG connectivity derived from the cross-spectra obtained with the same methods we have implemented, 2- include other methods for the comparison of MEG/EEG source spectra or cross-spectra, 3- perform the statistical comparison of the quality measures for the MEG/EEG spectra or cross-spectra obtained with different methods.


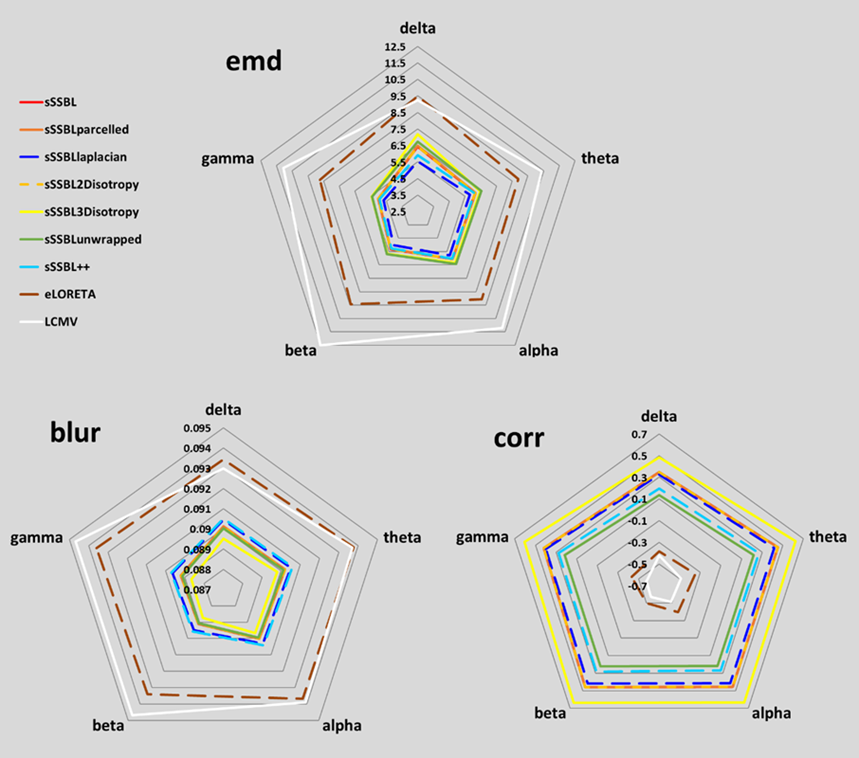
See an instance of the quality measures reported for the MEG of subject 175237 in Figure 11. We remind the reader that we measure leakage through the Spatial Dispersion (SD) of the PSF for the EEG solution (BLUR) and concurrency through the surface-based Earth Mover’s Distance (EMD) correlation (CORR) between the MEG-based and EEG-based spectra. Also, several different variants of sSSBL were explored to see the effect of different proposed structural priors. We remind the reader these priors incorporate different types of additional information (structural or functional) on the spectral activity. Namely sSSBL (no prior), sSSBLparcelled (cortical parcellation information), sSSBLlaplacian (Laplacian smooth operator), sSSBL2Disotropy (2D rotational invariance), sSSBL3Disotroopy (3D rotational invariance) and sSSBLunwrapped (surface curvature compensation). SSBL++.

Figure 11 Some quality measures for the source spectra of different bands computed for the outputs of the MEG/pseudoEEG simulation

Using our pipelines, we have produced consistent results in the comparison of the MEG/EEG populational spectral. The MEG and EEG were acquired in different populations conforming to the public databases: MEG of the Human Connectome Project.

# BC-VARETA ToolBox visual interfaces and configuration


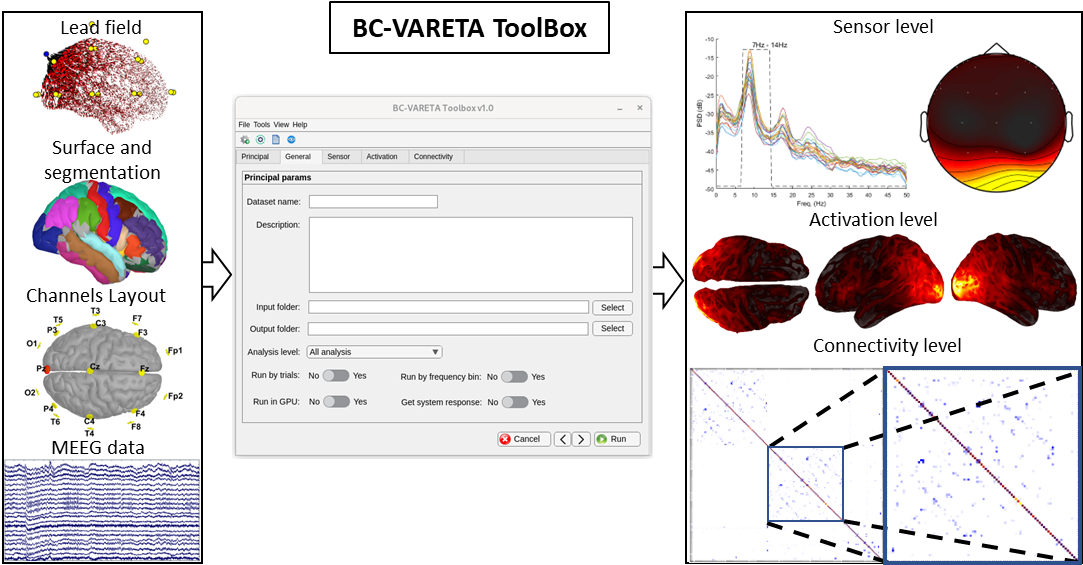
We have developed a suit based on MATLAB 2020 for the analysis of MEG/EEG data with BC-VARETA. Different tabs in the suit allow the selection of the level of analysis desired by the user, in hierarchical order 1) “sensor level”, MEG/EEG signal spectra, cross-spectral tensor, and spectral precision tensor 2) “activation level”, MEG/EEG source space spectra, 3) “connectivity level”, MEG/EEG source space cross-spectral tensor and spectral precision tensor. Note that the hierarchy in the “analysis levels” must be respected, and therefore, executing any level will be required to be feed with the outputs of the previous one.

Figure 12 General tab in the visual interface of the BC-VARETA Toolbox


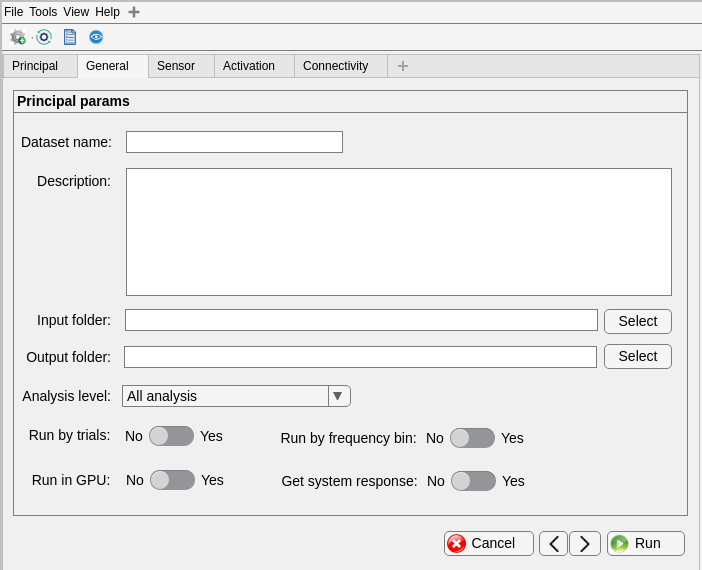
The general Tab 1) includes 1.1) the Dataset Name, 1.2) the Dataset description, 1.3) the field to locate the data input, and 1.4) the assigned output path for the results. The input data to BC-VARETA are the outputs of the head/source modeling pipeline (HCP_BST_source_head_modeler) in brainstorm format. Also include, the analysis at all levels 1.5) can executed in sequence and in different modes, which must be specified in the general parameters tab, which provide additional specificity of the estimators, such as: 1.6) analysis of independent trials of MEG/EEG data or data segments, 1.7) frequency-specific analysis of band analysis, 1.8) system response mode that allows the extraction of the source spectra and cross-spectral with a common sparse spatial support in the cortex for all frequencies. 1.9) that the user performs computation efficiently in GPU with the MATLAB CUDA libraries included in the BC-VARETA toolbox, which will still be saved in the background. The mode of the “activation” and “connectivity” levels will be tied to the configuration mode of the “sensor level.” Therefore, switching between modes will require the execution of the sensor level. See the tab options in Figure 13 and the description of parameters in Table 6

Figure 13 General params visual interface for BC-VARETA Toolbox

Table 6 BC-VARETA Toolbox general params description

| **General Tab** | | | | |
| --- | --- | --- | --- | --- |
| **Index** | **Name** | **Values** | **Default** | **Description** |
| 1 | General params | | | |
| 1.1 | Dataset name | Text | (empty) | The dataset name for the analysis |
| 1.2 | Description | text | (empty) | Dataset description |
| 1.3 | Input folder | Folder location | (empty) | Root folder location of the BC-VARETA structure. This folder should contain the subject structure for the analysis |
| 1.4 | Output folder | Folder location | (empty) | Root folder where the BC-VARETA Toolbox will export the results of the analysis |
| 1.5 | Analysis level |  | ‘All analysis’ | ‘All analysis’, ‘Sensor’, ‘Activation’, ‘Connectivity’, ‘Sensor and activation’, ‘Activation and connectivity’ |
| 1.6 | Run by trials | Yes or No | No | The time series of different trials are analyzed independently <<Yes>> or they are combined in a single time series <<No>>. |
| 1.7 | Run by frequency bin | Yes or No | No | Frequency components are analyzed independently <<Yes>> or according to a band-structured sparsity model corresponding to smoothness in the spectral domain <<No>> |
| 1.8 | Get system response | Yes or No | Yes | Decide whether the sources are responsive for all frequencies or not when applying sSSBL z-scores, <<No>> (apply z-scores independently for all frequencies) <<Yes>> (apply z-scores jointly for all frequencies) |
| 1.9 | Run in GPU | Yes or No | No | Run the tool using GPU |
| 3 | Cancel | | | |
| 4 | Run | | | |


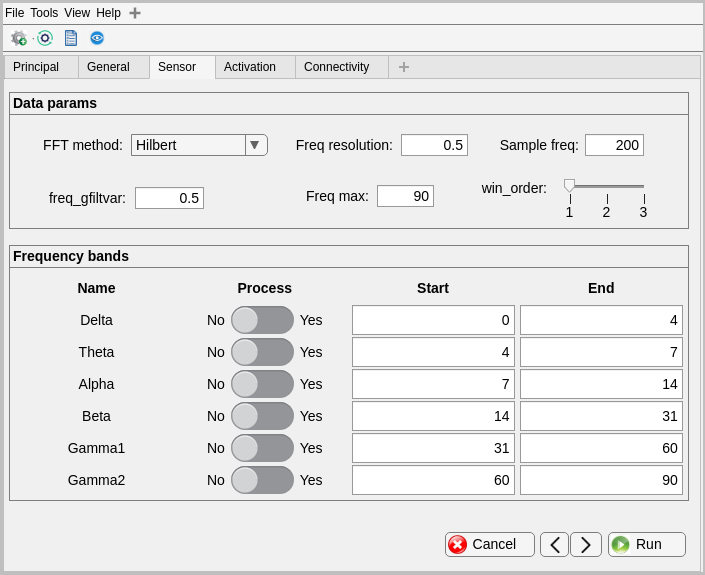
The “sensor level” Tab, see the graphics in Figure 14, contains options 1) for data params required for the spectral transformation of the MEG/EEG data. 1.1) FFT Method to compute the data Discrete Fourier Transform, 1.2) freq_resolution, the desired resolution of the frequency span in the spectral domain, which will be adjusted by selecting a specific size of the data segment to be taken to the spectral domain by means of the Discrete Fourier Transform, and at 1.3) a maximum frequency value given by the sampling frequency of the MEG/EEG data. 1.4) freq_gfiltvar, the variance of the Gaussian filter with the peak at every frequency spanning in the spectral domain.

Figure 14 Sensor level interface for the BC-VARETA Toolbox

The spectral analysis is based on the Hilbert envelope of filtered time series, obtained through the inverse Discrete Fourier Transform of the Fourier coefficients filtered with the Gaussian spectral response at the given frequency. This analysis is replicated for all frequencies 1.5), spanning up to a maximum Freq_max. 1.5) Spherical prolate windows are used to smooth the spectra even further, considering the number of windows that are defined in the variable win_order. Specification of the bands of frequency 1.6) and their limit must be provided by the user in case a nonstandard definition (different to those of an adult human); the activity in all bands will be analyzed according to the recommendations of the International Federation of Clinical Neurophysiology (IFCN) [71].

The bands of frequency are defined as follows. 2.1) delta 2.2)-2.3) [0Hz-4Hz], 2.4) theta 2.5)-2.6) [4Hz-7Hz], 2.7) alpha 2.8)-2.9) [7Hz-14Hz], 2.10) beta 2.11)-2.12) [14Hz-31Hz], 2.13) gamma1 2.14)-2.15) [31Hz-60Hz], 2.16) gamma2 2.17)-2.18) [60Hz-90Hz]. Table 7 describes all parameters of Figure 14.

Table 7 BC-VARETA Toolbox sensor level params description

| **Spectral Tab** | | | | |
| --- | --- | --- | --- | --- |
| **Index** | **Name** | **Values** | **Default** | **Description** |
| 1 | Data params | | | |
| 1.1 | FFT method |  | --Select-- |  |
| 1.2 | Freq resolution | 0.1 – 1 | 0.5 | Spectral resolution of the FFT |
| 1.3 | Sample freq | 100 – 600 | 200 | MEG/EEG sampling frequency |
| 1.4 | Freq gfiltvar | 0.1 – 1 | 0.5 | Variance (given in Hz) for the gaussian response filter of the data |
| 1.5 | Freq max | 20 – 90 | 90 | Maximum frequency under analysis, equal or higher than the ending of the faster band considered |
| 1.6 | Win order | 1 – 3 | 1 | Windowing Order, number of Slepian sequences in this case |
| 2 | Frequency bands | | | |
| 2.1 | Delta Process | Yes/No | Yes | delta band, the starting and ending frequencies of the band are <<f_start>> and <<f_end>>, set run in <<Yes>> if you want this band to be analyzed |
| 2.2 | Delta Start | 0 – 7 | 0 |  |
| 2.3 | Delta End | 0 – 7 | 4 |  |
| 2.4 | Theta Process | Yes/No | Yes | theta band, the starting and ending frequencies of the band are <<f_start>> and <<f_end>>, set run in <<Yes>> if you want this band to be analyzed |
| 2.5 | Theta Start | 2 – 9 | 4 |  |
| 2.6 | Theta End | 2 – 9 | 7 |  |
| 2.7 | Alpha Process | Yes/No | Yes | alpha band, the starting and ending frequencies of the band are <<f_start>> and <<f_end>>, set run in <<Yes>> if you want this band to be analyzed |
| 2.8 | Alpha Start | 6 – 15 | 7 |  |
| 2.9 | Alpha End | 6 – 15 | 14 |  |
| 2.10 | Beta Process | Yes/No | Yes | beta band, the starting and ending frequencies of the band are <<f_start>> and <<f_end>>, set run in <<Yes>> if you want this band to be analyzed |
| 2.11 | Beta Start | 12 – 35 | 14 |  |
| 2.12 | Beta End | 12 – 35 | 31 |  |
| 2.13 | Gamma1 Process | Yes/No | Yes | gamma1 band, the starting and ending frequencies of the band are <<f_start>> and <<f_end>>, set run in <<Yes>> if you want this band to be analyzed |
| 2.14 | Gamma1 Start | 29 – 65 | 31 |  |
| 2.15 | Gamma1 End | 29 – 65 | 60 |  |
| 2.16 | Gamma2 Process | Yes/No | Yes | gamma2 band, the starting and ending frequencies of the band are <<f_start>> and <<f_end>>, set run in <<Yes>> if you want this band to be analyzed |
| 2.17 | Gamma2 Start | 55 – 90 | 60 |  |
| 2.18 | Gamma2 End | 55 – 90 | 90 | 3.18 |
| 5 | Cancel |  |  |  |
| 6 | Run |  |  |  |


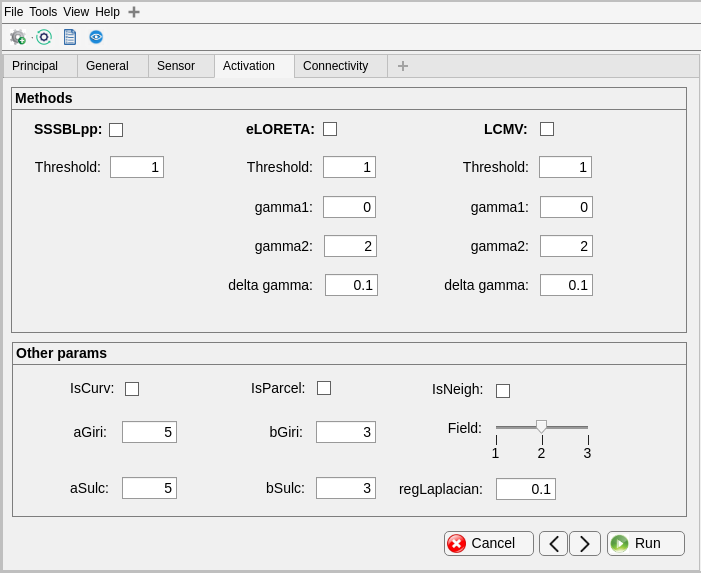
The “activation level” Tab, see the graphics in Figure 15, contains 1) the option to obtain the MEG/EEG source spectra from the sensor level cross-spectrum using different methods. 1.1) spectral Structured Sparse Bayesian Learning (sSSBL), the method revindicated by the BC-VARETA toolbox, with 1.2) statistical threshold for the mask of zeros of the source spectra that the user can select. Other methods are included, such as 1.3) eLORETA, with also 1.4) statistical threshold of the zero mask, regularization parameter gamma spanning in an interval 1.5) gamma1 to 1.6) gamma2 and 1.7) fineness delta_gamma; and 1.8) LCMV, also with analogous parameters to eLORETA 1.9) to 1.12). 2) Additional priors can be included to pursue the structured sparsity of the source spectra by means of 2.1) depth compensation based on the surface curvature. This type of priors unwraps the fields and hidden activation in cortical folds by deforming the lead field linearly to the curvature, with different linear coefficients 2.4)-2.5) for sulci and 2.6)-2.7) giri.

Figure *15* Activation level interface for the BC-VARETA Toolbox

This functionality takes effect for sSSBL, eLORETA, and LCMV 2.2). A mode of structured sparsity is explicitly defined for sSSBL through cortical parcellation, which is used as the basis for group penalization. 2.3) A mode of structured sparsity based on the cortical neighboring is introduced with the transformation of the Lead Fields by the deformed graph Laplacian, with deformation parameter 2.9) reg_Laplacian.

This functionality takes effect for sSSBL, eLORETA, and LCMV. 2.8) Finally, different modes of rotational invariance of the source orientation probabilities are introduced by the transformation of the lead fields regarding the cortical normal orientations, using 1) projected fields, 2) rationally invariant fields in cylindric coordinates regarding the normal orientations, and 3) rotationally invariant fields in the sphere. This functionality takes effect for sSSBL, eLORETA, and LCMV. A complete description of the parameters in Figure 15 is included in Table 8.

Table 8 BC-VARETA Toolbox activation level params description

| **Activation Tab** | | | | |
| --- | --- | --- | --- | --- |
| **Index** | **Name** | **Values** | **Default** | **Description** |
| 1 | Methods | | | |
| 1.1 | sSSBL | True or False | True | Run sSSBL method or not |
| 1.2 | Threshold | 0 – 1.73 | 1 | The threshold for the sSSBL++ F-scores, it can be set to <<1>> or higher (if <<1>> responses whose posterior variance is larger than the posterior mean is discarded) |
| 1.3 | eLORETA | True or False | True | Run eLORETA method or not |
| 1.4 | Threshold | 0 – 1.73 | 1 | The threshold for the eLORETA z-scores, it can be set to <<1>> or higher (if <<1>> responses whose posterior variance is larger than the posterior mean is discarded) |
| 1.5 | Gamma1 | 0 – 2 | 0 |  |
| 1.6 | Gamma2 | 0 – 2 | 2 |  |
| 1.7 | Delta gamma | 0.1 – 1 | 0.1 |  |
| 1.8 | LCMV | True or False | True | Run LCMV method or not |
| 1.9 | Threshold | 0 – 1.73 | 1 | The threshold for the LCMV z-scores, it can be set to <<1>> or higher (if <<1>> responses whose posterior variance is larger than the posterior mean is discarded) |
| 1.10 | Gamma1 | 0 – 2 | 0 |  |
| 1.11 | Gamma2 | 0 – 2 | 2 |  |
| 1.12 | Delta gamma | 0.1 – 1 | 0.1 |  |
| 2 | Other params | | | |
| 2.1 | IsCurv | True or False | True | Depth compensation factor for the Lead Field of giri and sulci <<false>> (no compensation) <<true>> (compensation) |
| 2.2 | IsParcel | True or False | True | Structured sparsity with smoothness of the responses within areas <<false>> (no smoothness) <<true>> (parcel smoothness) |
| 2.3 | IsNeigh | True or False | True | Structured sparsity with smoothness of the responses within areas <<false>> (no smoothness) <<true>> (parcel smoothness) |
| 2.4 | aGiri | 1 – 5 | 5 | Baseline of giri curvature factor |
| 2.5 | bGiri | 1 – 5 | 3 | Scale of giri curvature factor |
| 2.6 | aSulc | 1 – 5 | 5 | The baseline of sulci curvature factor is usually set to be higher than aSulc |
| 2.7 | bSulc | 1 – 5 | 3 | Scale of sulci curvature factor |
| 2.8 | Field | 1 – 3 | 2 | Use projected Lead Field or 3D profile with prior of rotational invariance <<1>> (projected Lead Field) <<2>> (2D field isotropy) and <<3>> (3D field isotropy) |
| 2.9 | regLaplacian | 0.1 – 1 | 0.1 |  |
| 3 | Cancel | | | |
| 4 | Start the process | | | |

The “connectivity level” Tab, see the graphics in Figure 3-12, contains options 1) to obtain the MEG/EEG source cross-spectral tensor and the spectral precision tensor from the sensor level cross-spectrum using different methods. 1.1) the Hidden Gaussian Graphical State-Space Model, one of the methods revindicated by the BC-VARETA toolbox, with sparse precision tensor obtained by mean of the Hermitian Graphical LASSO (hgLASSO) 1.2) hgLASSO estimator that is obtained following determination of the source cross-spectral tensor by means of sSSBL, eLORETA, and LCMV. Threshold for the mask of zeros of the source spectra that the user can select. 2) Additional priors can be included to pursue the structured sparsity of the source spectral precision tensor. Analogously to the activation level, by means of 2.1) depth compensation based on the surface curvature, this type of priors unwraps the fields and hidden activation in cortical folds by deforming the lead field in linear proportion to the curvature, with different linear coefficients 2.4)-2.5) for sulci and 2.6)-2.7) for giri. 2.2) A mode of structured sparsity that is based on the cortical neighboring is introduced with the transformation of the Lead Fields by the deformed graph Laplacian, with deformation parameter 2.17) reg_Laplacian. 2.11) Modes of rotational invariance of the source orientation cartesian product probabilities are introduced by transforming the lead fields regarding the cortical normal orientations, as in the activation level. Specific parameters for HIGGS and hgLASSO must be configured. 2.10) the data noise interior thresholds that shall be considered in HIGGS EM computations. 2.11) the lower and upper limit thresholds upon the Rayleigh distribution of the spectral precisions determined by hgLASSO from the peak of the distribution 2.13) rth1 to the tail 2.14) rth2. 2.16) eigreg, the regularization parameters of the eigenvalues for matrices computing within HIGGS and hgLASSO iterations. 2.3) The user can set the Prewarming that is included as a step anticipating EM computations by HIGSS. This is based on the initialization of the source cross-spectral tensor by means of sSSBL. 2.8) the maximum number of iterations for HIGGS EM loop, and 2.9) the maximum number of iterations for the hgLASSO LQA loop. 2.12) the type of penalty used in the computation of the source precision tensor via the prior free estimator val=0, the hgLASSO estimator val=1, or the Hermitian Graphical Ridge val=2. 2.15) Finally, the number of EM iterations forward to obtain the optimal threshold of the Rayleigh distribution, which is evaluated by the trend of the EM global likelihood. A complete description of the
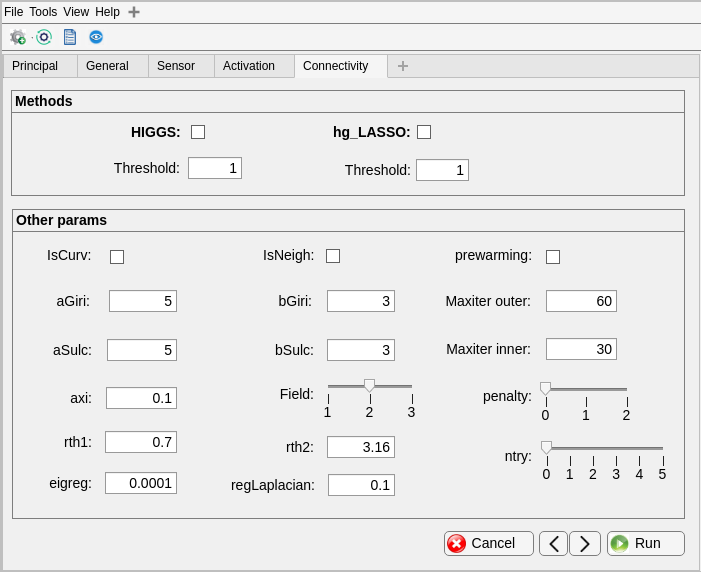
parameters in Figure 11 is included in Table 9.

Figure *11* Connectivity level interface for the BC-VARETA Toolbox

Table 9 BC-VARETA Toolbox connectivity level params description

| **Connectivity Tab** | | | | |
| --- | --- | --- | --- | --- |
| **Index** | **Name** | **Values** | **Default** | **Description** |
| 1 | Methods | | | |
| 1.1 | HIGGS | True or False | True | Run HIGGS method or not |
| 1.2 | Threshold | 0 – 1.73 | 1 | The threshold for the HIGGS F-scores, it can be set to <<1>> or higher (if <<1>> responses whose posterior variance is larger than the posterior mean is discarded) |
| 1.3 | HG_LASSO | True or False | True | Run HG_LASSO or not |
| 1.4 | Threshold | 0 – 1.73 | 1 | The threshold for the HG_LASSO F-scores, it can be set to <<1>> or higher (if <<1>> responses whose posterior variance is larger than the posterior mean is discarded) |
| 2 | Other params | | | |
| 2.1 | IsCurv | True or False | True | Depth compensation factor for the Lead Field of giri and sulci <<No>> (no compensation) <<Yes>> (compensation) |
| 2.2 | IsNeigh | True or False | True | no neighbor structure <<No>>, Laplacian neighbor structure <<Yes>>. |
| 2.3 | prewarming | True or False | True | Perform prewarming of the HIGGS EM by means of a two-step method (first activation and then connectivity), <<0>> (no prewarming) <<1>> (does prewarming) |
| 2.4 | aGiri | 1 – 5 | 5 | Baseline of giri curvature factor |
| 2.5 | bGiri | 1 – 5 | 3 | Scale of giri curvature factor |
| 2.6 | aSulc | 1 – 5 | 5 | Baseline of sulci curvature factor, usually is set to be higher than aSulc |
| 2.7 | bSulc | 1 – 5 | 3 | Scale of sulci curvature factor |
| 2.8 | Maxiter outer | 10 – 60 | 60 | Maximum number of iterations for the HIGGS EM outer loop |
| 2.9 | Maxiter inner | 10 – 60 | 30 | Maximum number of iterations for the HIGGS hg_LASSO inner loop |
| 2.10 | axi | 0.1 – 1 | 0.1 | Instrumental noise inferior limit that can be defined according to experimental information, <<1E-1>> represents 10% |
| 2.11 | Field | 1 – 3 | 2 | Use projected Lead Field or 3D profile with prior of rotational invariance <<1>> (projected Lead Field) <<2>> (2D field isotropy) |
| 2.12 | Penalty | 0 – 2 | 1 | Penalization model for HIGGS  <<0>> (naive or vareta)  <<1>> (Hermitian graphical LASSO or hg_LASSO)  <<2>> (Hermitian graphical Ridge or hgRidge),  if set to <<1>> the computational cost is higher but with full statistical guaranties,  <<2>> does not offer full guaranties but can be very similar to <<1>> |
| 2.13 | Rth1 | 0.1 – 1 | 0.7 | Inferior Rayleigh threshold <<0.7>> is the maximum of the Rayleigh distribution |
| 2.14 | Rth2 | 0.1 – 5 | 3.16 | Superior Rayleigh threshold <<3.16>> is the 99% percentile of the Rayleigh distribution |
| 2.15 | ntry | 0 – 5 | 5 | Number of iterations forward in evidence HIGGS evidence optimality prediction to evaluate the performance of the hg_LASSO Rayleigh threshold at every iteration |
| 2.16 | eigreg | 0.0001 – 0.01 | 0.0001 |  |
| 2.17 | regLaplacian | 0.1 – 1 | 0.1 |  |
| 3 | Cancel | | | |
| 4 | Run | | | |
